# Mechanical Stimulation Induces Yap Mediated OCTN2 Transcription to Enhance Carnitine Metabolism in Sarcopenia

**DOI:** 10.1002/jcsm.70052

**Published:** 2025-09-17

**Authors:** Yahong Lu, Yu Bai, Weiqing Li, Zhiguo Zhou, Heihuan Lai, Xingyu Hu, Tao Yang, Chendi Wang, Yitao Chen, Keping Gan, Kechi Li, Haiwei Ma, Lin Shen, Dengwei He

**Affiliations:** ^1^ The Fifth Affiliated Hospital of Wenzhou Medical University, Lishui Municipal Central Hospital Lishui Zhejiang Province China; ^2^ Orthopedic Department Taizhou Hospital Affiliated to Wenzhou Medical University, Linhai, China; Enze Medical Research Centre, Taizhou Hospital Affiliated to Wenzhou Medical University Linhai China

**Keywords:** carnitine, fatty acids, lipid deposition, mechanical force, mitochondrial dysfunction, muscular atrophy, OCTN2, Yap/Tead4

## Abstract

**Background:**

Sarcopenia is a systemic skeletal muscle disease that seriously affects the health of the aged population. Exercise prevents sarcopenia, but the underlying mechanobiological and metabolic mechanisms need to be further investigated.

**Methods:**

Carnitine and organic cation transporter 2 (OCTN2) levels were assessed in humans and animals with sarcopenia. Skeletal muscle function and histomorphology were assessed in an animal model. Mitochondrial structure and function were assessed via MitoSox and JC‐1 staining, seahorse assays and electron microscopy. Molecular mechanisms were assessed by Western blot analysis, qPCR, a luciferase reporter gene assay, chromatin immunoprecipitation and immunofluorescence in C2C12 myotubular cells.

**Results:**

A total of 66 patients were included in the study (Healthy group, % females: 44.74%, mean age: 67.40 ± 8.2, mean BMI: 24.7 ± 3.80 kg/m^2^; Sarcopenia group, % females: 39.29%, mean age: 71 ± 8.42, mean BMI: 23.1 ± 2.98 kg/m^2^). Serum carnitine levels decreased in sarcopenia patients (10 868 ± 3466 ng/mL vs. 8469 ± 2360 ng/mL, *p* < 0.01). Carnitine is an independent protective factor for sarcopenia (OR, 0.757; 95% CI 0.599–0.923, *p* = 0.0107). Carnitine and OCTN2 levels also decreased in the muscles of mice with dexamethasone‐induced muscle atrophy (carnitine: −16.5%, *p* < 0.05) and aged mice (carnitine: −32.03%, *p* < 0.01). Suppressed expression of OCTN2 led to a decrease in muscle carnitine (2983 ± 466.3 ng/mL vs. 2517 ± 355.3 ng/mL, *p* < 0.05), as well as muscle atrophy in mice. Swimming exercise enhanced mice carnitine‐dependent fatty acid oxidation and increased OCTN2 expression (OCTN2: +8.4%, *p* < 0.05). Knockdown of OCTN2 partially reduced this effect during swimming. Cellular experiments revealed that mechanical stimulation upregulated OCTN2 expression. OCTN2 knockdown impaired myotube formation and led to the disruption of the cellular mitochondrial structure. Further mechanistic studies showed that mechanical forces enhanced OCTN2 transcription and regulated carnitine metabolic homeostasis through the Yap/Tead4 pathway. Yap agonist XMU alleviated dexamethasone‐induced muscle atrophy (grip: +13%, *p* < 0.05; cross‐sectional area of the gastrocnemius muscle: +8%, *p* < 0.05). In a high‐fat diet mouse model and in cellular experiments, carnitine supplement improved mitochondrial structure and alleviated mitochondrial dysfunction by reducing excessive lipid accumulation and thus altered myocyte fate.

**Conclusion:**

Swimming and carnitine supplementation alleviated sarcopenia. The mechanism was closely related to the enhancement of OCTN2 expression after Yap activation and the enhancement of carnitine‐mediated lipid metabolism. These findings reveal exercise regulates skeletal muscle by coupling mechanics and metabolism synergetically. We provide a new therapeutic strategy for sarcopenia.

## 1 Introduction

Sarcopenia presents as a widespread reduction in muscle fibres and reduced skeletal muscle function. This degenerative loss of muscle leads to decreased athletic ability in the aging process [1, 2]. Sarcopenia is also associated with diseases such as obesity, cirrhosis and glucocorticoids use [3, 4, 5]. Currently, drugs for the treatment of sarcopenia are still under development and have limited efficacy [6, 7]. Therefore, identifying new therapeutic targets for sarcopenia is particularly important.

Carnitine is an amino acid derivative that exists in biological organisms in the form of free carnitine and acyl conjugates. The carnitine concentration varies greatly among tissues, with skeletal muscle being the most carnitine‐rich tissue, accounting for more than 90% of the total carnitine content within the human body [8]. The most important physiological role of carnitine is the transport of impermeable long‐chain fatty acids (more than 12 carbons) into the mitochondria for *β*‐oxidation energy supply. This process is critical to mitochondrial function. Previous studies by our research team have demonstrated the importance of carnitine in osteoporosis [9], but the specific role of carnitine in sarcopenia is unknown. Because only the liver, kidneys and brain have the full set of enzymes to synthesise carnitine actively, other tissues rely on active transport to take up carnitine from the blood. This is especially true for tissues such as cardiac muscle and skeletal muscle, which are highly dependent on the energy produced by *β*‐oxidation. Tissue uptake of carnitine is dependent on the specific carnitine transporter channel protein OCTN2. Primary carnitine deficiency (PCD, OMIM 212140) results when mutations occur in the SLC22A5 gene encoding OCTN2. PCD is an autosomal recessive metabolic disease in which patients have decreased carnitine levels and present with skeletal muscle disorders such as skeletal muscle weakness, polymyositis and complications such as fatty liver and dilated cardiomyopathy [10]. This rare disease illustrates the importance of OCTN2 and carnitine in the skeletal muscle system. Therefore, our initial goal was to investigate the correlation between OCTN2/carnitine and sarcopenia.

The mechanism of sarcopenia is complex. Previous studies have shown that the age‐related fat infiltration of skeletal muscle is strongly associated with muscle atrophy (sarcopenia) [11, 12]. These ectopically deposited lipids and their metabolic secondary products, such as diacylglycerol (DAG), lead to skeletal muscle insulin resistance, which in turn interferes with the normal metabolism of skeletal muscle cells. In addition, local excessive free fatty acids can promote the secretion of inflammatory factors and other senescence‐associated secretory phenotypes (SASPs) in muscle tissue, significantly impairing mitochondrial function and generating excessive ROS, forcing skeletal muscle into a vicious insulin resistance–inflammation cycle and accelerating skeletal muscle degeneration, ultimately leading to sarcopenia [13]. Additionally, mitochondrial dysfunction is involved in the development of sarcopenia [14, 15, 16]. Does carnitine, one of the key molecules of mitochondrial fatty acid metabolism, play a role in this process and thus influence muscle cell fate? This question has yet to be answered.

Exercise is closely linked to the muscular system and numerous studies have shown that mechanical stimulation plays an important role in improving the health of skeletal muscle [17, 18, 19]. Physically active people tend to have greater muscle strength, which is associated with the repeated mechanical force stimulation of muscle tissue [20]. Mechanical stimulation is a key physical factor in the alteration of the cellular environment. Many signalling pathway effectors, such as YAP/TAZ, Piezo1, Wnt/*β*‐catenin and Rho/ROCK, are regulated by mechanistic signalling and have an impact on skeletal muscle physiology [21, 22, 23, 24]. Exercise is also a key factor in energy balance, which involves changing fat content to reduce the incidence of chronic diseases such as obesity [25]. Notably, the metabolism of carnitine is affected by mechanical forces. During energy expenditure (exercise), the free carnitine demand increases, and free carnitine binds to long‐chain fatty acids and is converted to acylcarnitine; thus, acylcarnitine levels increase [26]. OCTN2 is a channel for carnitine to enter the cell. Therefore, our second goal was to investigate whether OCTN2‐mediated carnitine content responds to the regulatory effects of mechanical forces in the muscular system.

## 2 Materials and Methods

### 2.1 Human Subjects

This retrospective study was approved by the Ethics Committee of Lishui Central Hospital, Zhejiang Province (approval number 2024‐115). Venous blood was collected from the elbow vein of each patient between 6 AM and 8 AM, centrifuged and stored at −80°C. Written informed consent was obtained from all patients.

According to the Asian Working Group on Sarcopenia 2019 (AWGS 2019), we grouped patients primarily based on grip strength (Male: < 28 kg, Female: < 18 kg to sarcopenia group), skeletal muscle index (SMI) assessed by DXA (Male: < 7.0 kg/m^2^, Female: < 5.4 kg/m^2^ to sarcopenia group), and the degree of fat infiltration assessed by MRI. MRI was used to assess fat infiltration and to assist in defining sarcopenia. The degree of infiltration was classified according to the Goutallier classification system, which is a semiquantitative visual assessment method. The classification criteria are detailed in Table S5. We categorised patients with Goutallier classification results of Grade 3 and 4 into the sarcopenia group. Details of the equipment used for MRI and DXA are shown in Table S6.

### 2.2 Experimental Animals

C57BL/6 J mice at 6–9 weeks and 20 months of age were purchased from HangSi Biotech (Hangzhou, China). All the animal experiments were approved by the Lishui Central Hospital Ethics Committee (approval number 2024YD0162). All the mice were kept in specific‐pathogen‐free (SPF)‐grade animal facilities. All experiments were performed following the principles of the Animal Treatment Guidelines and the Guide for the Care and Use of Laboratory Animals (NIH, Bethesda, MD).

### 2.3 Statistical Analysis

Each experiment was performed three times and similar results were obtained. Data from two groups or more than two groups were analysed via Student's *t* test or one‐way ANOVA, respectively. The results of the study are expressed as the mean ± standard deviation (SD). All the data were analysed using GraphPad Prism 8.0. *p* < 0.05 was considered statistically significant.

Additional details are available in the online Suporting Information.

## 3 Results

### 3.1 Decreased OCTN2 and Carnitine Levels in Patients and Mice With Sarcopenia

**FIGURE 1:**
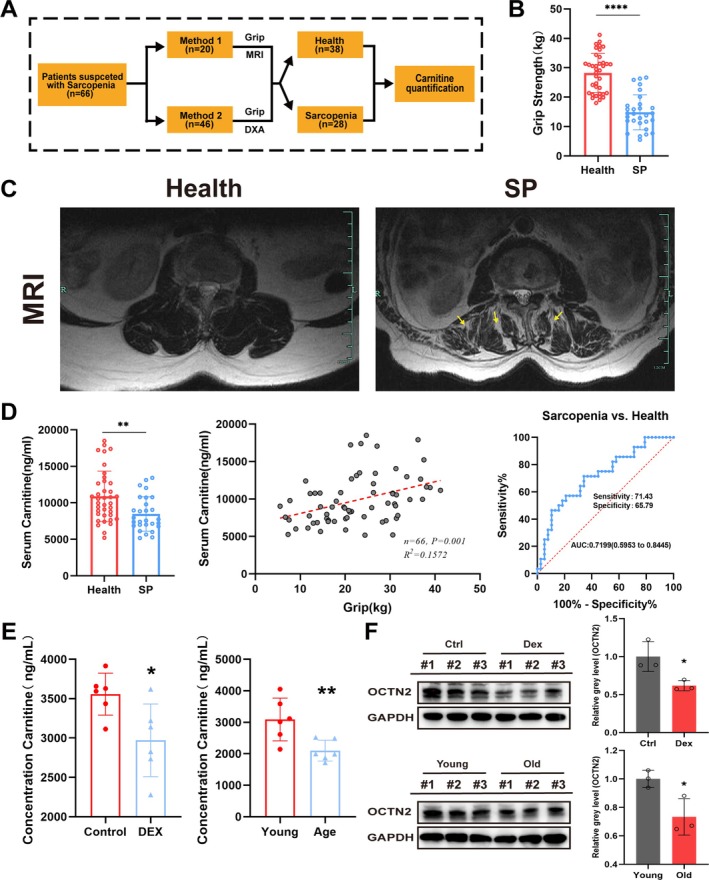
Dysregulation of OCTN2 and carnitine levels in patients and mice with sarcopenia. (A) Schematic of the retrospective analysis of the serum carnitine concentration in patients with sarcopenia. (B) Grip strength levels of sarcopenia patients and control patients. (C) Typical MR images of the psoas major muscle in non‐sarcopenia patients and sarcopenia patients. (D) Serum carnitine content in sarcopenia patients and control patients (left); Pearson correlation analysis between canitine level and grip (mid); ROC curves of carnitine distinguishing between SP patients and relatively healthy patients (right). (E) Carnitine levels in the muscles of mice with DEX‐induced sarcopenia and control mice as well as in the muscles of senescent mice and young mice. (F) OCTN2 protein levels were analysed by Western blot in the muscles of mice with DEX‐induced sarcopenia and control mice as well as in the muscles of senescent mice and young mice. The intensity of the OCTN band relative to that of the GAPDH band was quantified via ImageJ software (*n* = 3). *p < 0.05, **p < 0.01, ***p < 0.001, ****p < 0.0001, means ± SDs.

As shown in Figure 1A, patients were grouped by grip strength, MRI, or DXA into the sarcopenia group and healthy control groups (Figure 1B,C). There was no significant difference in patient age, sex, height, or weight (Figure S1A). The serum level of carnitine in the sarcopenia group was significantly lower than that in the healthy control group (8469 ± 2360 ng/mL vs. 10 868 ± 3466 ng/mL, *p* < 0.01; Figure 1D). Further statistical analysis showed that, when adjusted for sex, age and BMI, carnitine was an independent protective factor for sarcopenia (OR, 0.757; 95% CI 0.599–0.923, *p* = 0.0107; Table S1). According to Pearson's correlation analysis, serum carnitine levels were positively correlated with grip strength (Figure 1D). ROC curves showed that carnitine levels could distinguish SP patients from relatively healthy patients with good sensitivity and specificity (Figure 1D). DEX‐induced mice muscle atrophy was compared with control mice. Muscle function tests and H&E staining confirmed the successful establishment of the muscle atrophy model (Figure S1B–D). The muscle carnitine content of DEX‐induced muscle atrophy model mice was 2971 ± 462.1 ng/mL, which was significantly lower than that of control mice (3558 ± 267 ng/mL) (*p* < 0.05; Figure 1E). In addition, 9‐week‐old mice and 20‐month‐old mice were also compared. The results revealed a reduction in the carnitine content in aged mouse muscle (3089 ± 677.9 ng/mL vs. 2100 ± 332 ng/mL, *p* < 0.01; Figure 1E). Moreover, western blot results revealed that OCTN2 expression decreased in the muscles of muscle atrophy model mice and aged mice (Figure 1F). These results suggest that carnitine and OCTN2 are downregulated under sarcopenic conditions.

### 3.2 OCTN2 Deficiency Decreases the Carnitine Content, Contributing to Sarcopenia Progression

**FIGURE 2:**
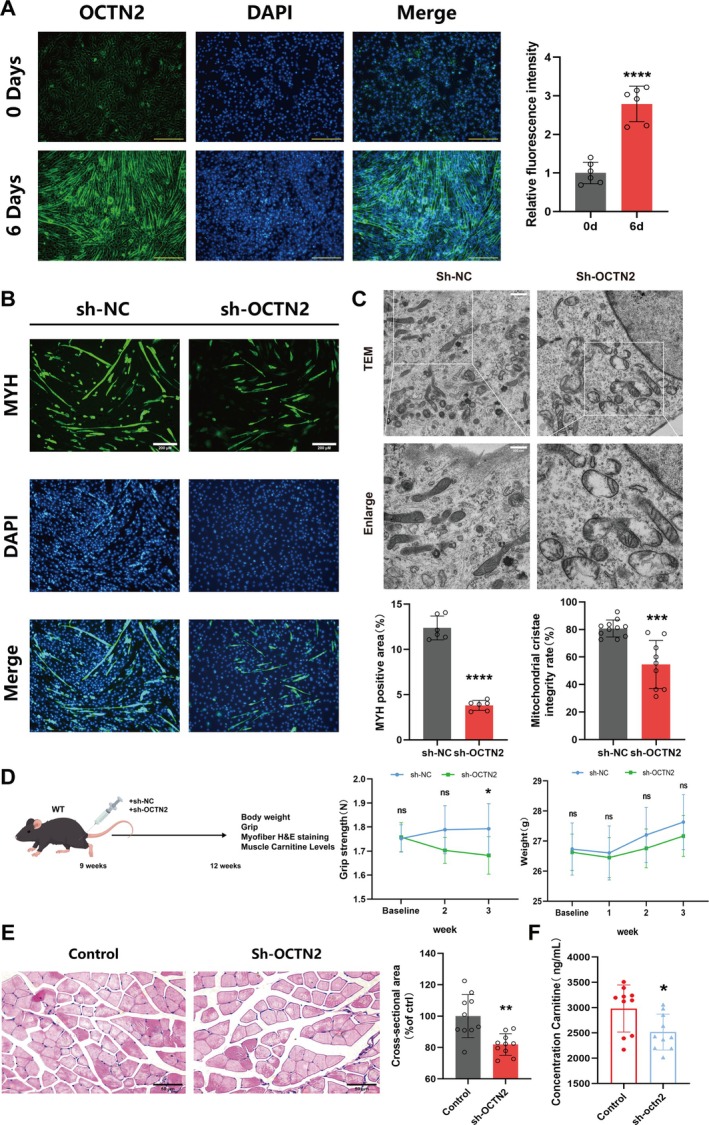
OCTN2 deficiency decreases the carnitine content, contributing to sarcopenia progression. (A) Cellular immunofluorescence was performed to assess OCTN2 expression in myotubes on day 0 versus day 6 of differentiation and quantitative fluorescence intensity analysis was performed; scale bar = 250 μm. (B) Representative images of MYH immunofluorescence in C2C12 myotubes transfected with shOCTN2 or shNC. Quantification of the MHC‐positive area/total area via ImageJ software. (C) Mitochondrial morphological changes in C2C12 myotubes transfected with shOCTN2 or shNC were observed via transmission electron microscopy. Quantitative statistics of the proportion of mitochondria with an intact crista structure to total mitochondria in each cell. (D) Experimental design model (left); Grip strength and body weight were measured for mice treated with adenoviral sh‐OCTN2 for 3 weeks (right). (E) H&E staining of mouse gastrocnemius muscle and statistics of the cross‐sectional area of sections. (F) Carnitine levels in the muscles of shOCTN2‐treated mice and shNC‐treated mice. *p < 0.05, **p < 0.01, ***p < 0.001, ****p < 0.0001, means ± SDs.

To explore the relationships between OCTN2, carnitine and sarcopenia, the expression levels of OCTN2 mRNA and protein in C2C12 myoblasts were investigated. OCTN2 expression was upregulated during myogenic differentiation (Figure S2A,B), a finding that was confirmed by cellular immunofluorescence experiments (Figure 2A). Cellular immunofluorescence revealed that myotube differentiation was significantly impaired in cells with OCTN2 silencing (Figure 2B). Biotransmission electron microscopy revealed lower mitochondrial crista density and membrane rupture in cells expressing sh‐OCTN2 than in cells expressing shNC, indicating a damaged and abnormal state (Figure 2C). Next, C57BL6 mice injected with adenovirus‐mediated sh‐OCTN2 or shNC in the hind limbs were compared (Figure 2D). Western blot results revealed successful OCTN2 knockdown (Figure S2C). The grip test results revealed a decrease in muscle strength in sh‐OCTN2 mice (1.793 ± 0.1045 N vs. 1.682 ± 0.078 N, *p* < 0.05), but there was no significant difference in body weight (Figure 2D). H&E staining of mouse gastrocnemius muscle revealed that the muscle fibre cross‐sectional area was significantly smaller in sh‐OCTN2‐treated mice than in control mice (Figure 2E). Moreover, muscle carnitine content was reduced (2983 ± 466.3 ng/mL vs. 2517 ± 355.3 ng/mL, *p* < 0.05; Figure 2F) and muscle fatty acid oxidising activity was decreased (0.1607 ± 0.026 U/gprot vs. 0.04 ± 0.013 U/gprot, *p* < 0.0001; Figure S2D) after knockout of OCTN2 in mice. In conclusion, these results indicate that OCTN2 plays an important role in skeletal muscle and that OCTN2 deficiency causes muscle atrophy.

### 3.3 OCTN2 Expression Occurs in Response to Mechanical Force Through the Hippo/YAP/TEAD4 Signalling Pathway

**FIGURE 3:**
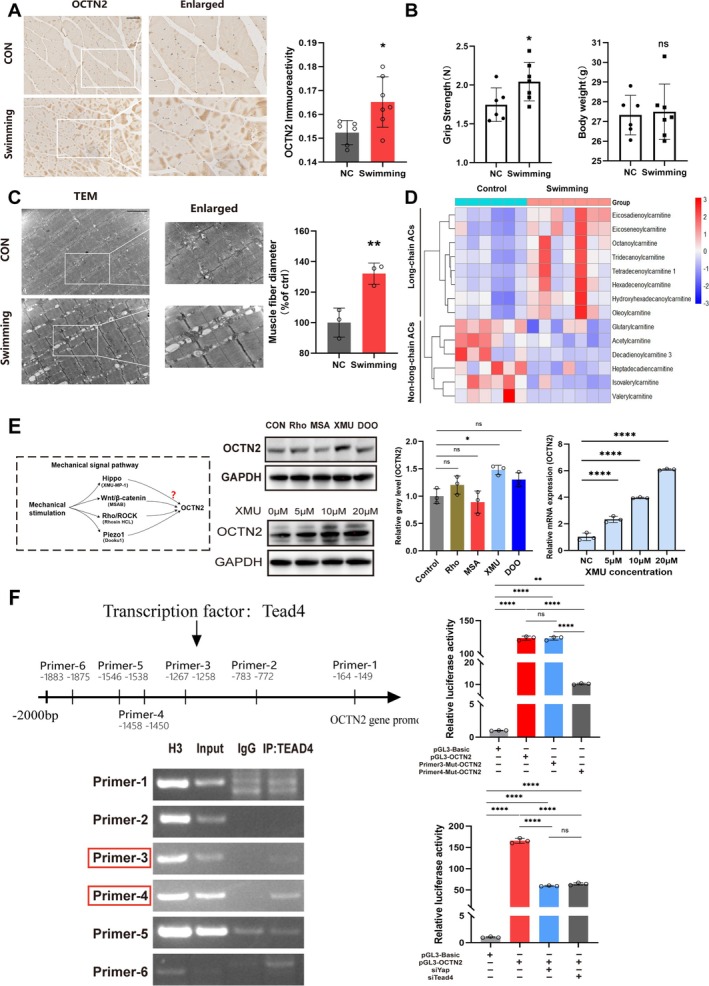
OCTN2 expression occurs in response to mechanical force through the Hippo/YAP/TEAD4 signalling pathway. (A) Immunohistochemical analysis of OCTN2 immunoreactivity in swimming mice (*n* = 7) versus WT mice born in the same litter (*n* = 6); scale bar = 50 μm. (B) The body weights and grip strengths of the mice were recorded and measured after 2 months of swimming. (C) Mouse muscle fibres were observed via transmission electron microscopy and muscle fibre diameters were measured. (D) Metabolomics of muscle tissues from swimming mice and control mice. (E) C2C12 myotubes were treated with Rhosin HCL, MSAB, XMU‐MP‐1 or Dooku1 alone for 24 h. OCTN2 protein expression was assessed via Western blot. C2C12 myotubes were treated with different concentrations of XMU‐MP‐1 for 24 h. OCTN2 protein expression was assessed via Western blot. The mRNA expression of OCTN2 was assessed via RT–qPCR. (F) ChIP analysis of Tead4 aggregates on the OCTN2 promoter region (left). Relative luciferase activity was measured in cell lysates to quantify the gene expression of OCTN2 in C2C12 cells (right). *p < 0.05, **p < 0.01, ***p < 0.001, ****p < 0.0001, means ± SDs.

Exercise is closely linked to the muscular system, and studies have shown a strong link between exercise and carnitine metabolic status [26]. The changes in OCTN2 expression in exercised mice were examined. Mice were subjected to swimming exercise (Figure S3A); immunohistochemistry revealed that OCTN2 expression was upregulated in exercised mice (Figure 3A). The grip strength test revealed greater grip strength in exercised mice (1.747 ± 0.216 N vs. 2.044 ± 0.249 N, *p* < 0.05), with no significant difference in body weight between exercised and control mice (Figure 3B). Electron microscopy of gastrocnemius muscle tissue revealed greater muscle fibre diameters in exercised mice than in control mice (Figure 3C). Moreover, metabolomics results of targeted lipid and acylcarnitine profiles in exercised and nonexercised mice revealed significant differences in acylcarnitine analogues, with short‐ and medium‐chain acylcarnitines being reduced. In contrast, the levels of long‐chain acylcarnitines, which are metabolites of long‐chain fatty acids that cannot pass through the mitochondrial membrane alone, were increased (Figure 3D). The above experimental results demonstrate that mechanical stimulation enhances muscle mass, increases OCTN2 expression and promotes the carnitine‐mediated metabolism of long‐chain fatty acids.

Next, the specific molecular mechanisms by which mechanical forces regulate OCTN2 were explored. Various mechanical pathways, including the Hippo, Piezo1, Wnt/*β*‐catenin and Rho/ROCK pathways, are investigated. Hippo/Yap agonist XMU but not other signalling pathways, significantly upregulated OCTN2 expression. OCTN2 mRNA and protein expression increased in a dose‐dependent manner in response to the Yap agonist XMU (Figure 3E). Therefore, we focused on YAP. 5% tension stimulation was applied to C2C12 myotubes via the CELL TALK Mechanical Cell Strainer. Tension activated the YAP pathway and increased OCTN2 expression (Figure S3B), whereas the inhibition of Yap resulted in a decrease in OCTN2 expression under tension stimulation (Figure S3C).

Using biological information analysis, we did not find any binding sites for Yap in the OCTN2 promoter region. However, previous studies have shown that TEAD4, a downstream regulator of Yap, can promote myogenic differentiation [27]. Other studies have shown that Yap interacts with TEAD transcription factors to regulate skeletal muscle mass and protein synthesis [28]. We discovered that the OCTN2 promoter region includes multiple binding sites for TEAD4 (Figure 3F). As a cotranscription factor, YAP is usually coupled to TEAD proteins to initiate transcriptional events. Therefore, we hypothesised that YAP is involved in the regulation of OCTN2 at the transcription level through TEAD4. Chromatin immunoprecipitation (CHIP) experiments revealed that TEAD4 bound to the OCTN2 promoter region (Figure 3F) and that the binding site was most likely at‐1258/‐1267 versus‐1450/‐1458. To validate those findings, these two fragments were mutated and inserted into the pGL4‐basic vector separately and then transfected into C2C12 myoblasts for a dual‐luciferase gene reporter assay to test the activities of the unmutated and mutated fragments. Transfection of the wild‐type and 1258/‐1267 site‐mutated OCTN2 promoter regions enhanced luciferase activity, but the mutation at the‐1450/‐1458 site in the OCTN2 promoter region did not affect luciferase activity (Figure 3F). On the basis of these findings, we considered the‐1450/‐1458 region as the binding site of TEAD4 to OCTN2. Moreover, a luciferase reporter gene assay demonstrated that silencing Tead4 or Yap reduced the transcriptional activity of OCTN2 (Figure 3F). In summary, these findings indicate that mechanical forces induce the transcriptional activation of OCTN2 via the YAP/TEAD4 pathway.

### 3.4 OCTN2 Deficiency Attenuates the Response to Mechanical Forces and Enhancing YAP With XMU Alleviates Muscle Atrophy

**FIGURE 4:**
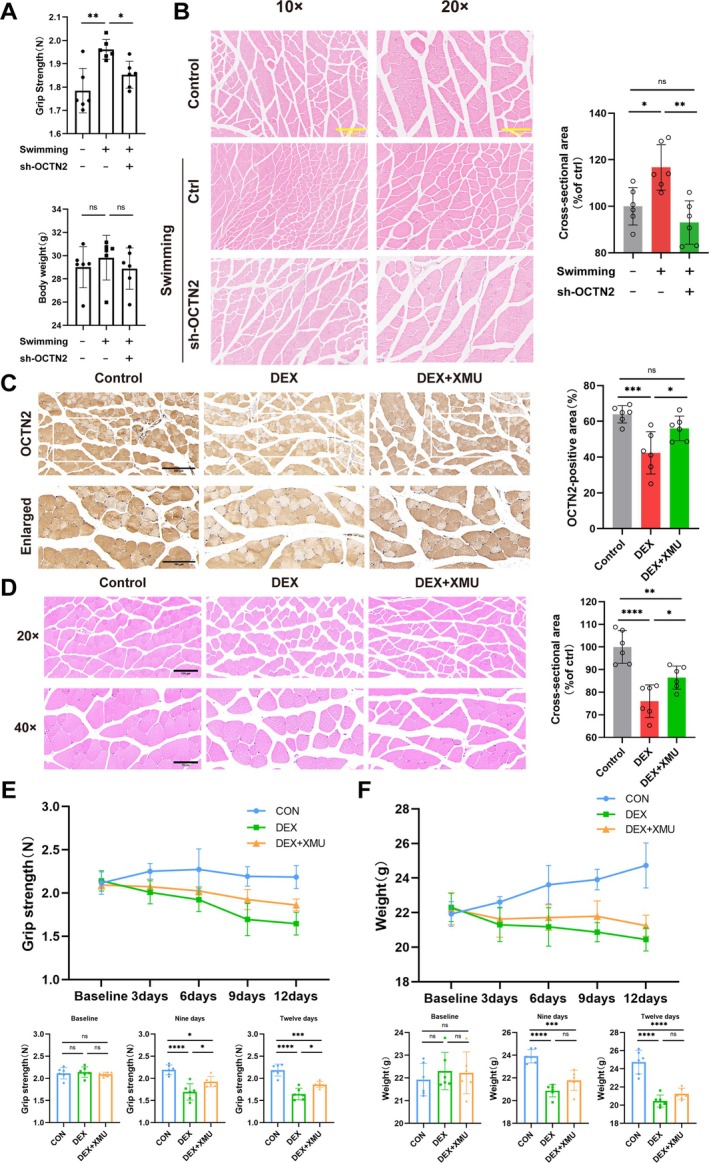
OCTN2 deficiency attenuates the response to mechanical forces and enhancing YAP with XMU improves muscle atrophy. (A) Grip strength and body weight of the mice. (B) H&E staining of the mouse gastrocnemius muscle and measurement of the cross‐sectional area. (C) Immunohistochemical analysis of mouse OCTN2 immunoreactivity. (D) Representative H&E staining of cross‐sections of the mouse gastrocnemius muscle and the corresponding cross‐sectional area. (E) Grip strength of the mice. (F) Body weights of the mice. *p < 0.05, **p < 0.01, ***p < 0.001, ****p < 0.0001, means ± SDs.

To determine whether OCTN2 is regulated by mechanical forces, the mice injected with sh‐OCTN2 adenovirus were subjected to swimming exercise (Figure S4A). Exercise increased grip strength (1.784 ± 0.095 N vs. 1.961 ± 0.042 N, *p* < 0.01) with respect to muscle fibre cross‐sectional area in mice, but the absence of OCTN2 attenuated this beneficial effect (1.961 ± 0.042 N vs. 1.853 ± 0.058 N, *p* < 0.05; Figure 4A,B). Next, the therapeutic effects of OCTN2/carnitine on sarcopenia were investigated (Figure S4B). The immunohistochemistry results revealed a decrease in OCTN2 expression in the DEX group, whereas XMU treatment increased OCTN2 expression (Figure 4C). DEX‐treated mice presented decreased grip strength (2.184 ± 0.134 N vs. 1.645 ± 0.131 N, *p* < 0.0001), body weight (24.73 ± 1.298 g vs. 20.45 ± 0.665 g, *p* < 0.0001) and muscle fibre cross‐sectional area but the administration of XMU restored the muscle fibre cross‐sectional area and grip strength (1.645 ± 0.131 N vs. 1.862 ± 0.069 N, *p* < 0.05) of the mice; body weight also tended to increase, but the change was not significant (Figure 4D–F). Overall, these results suggest that OCTN2 deficiency in muscle results in a weakened muscle reaction to mechanical forces and that the administration of the YAP agonist XMU ameliorates muscle atrophy.

### 3.5 Carnitine Reduces Lipid Accumulation Caused by Excess Fatty Acids

Next, how carnitine specifically affects the fate of muscle cells was explored. As previously described, impaired lipid metabolism is one of the causes of the progression of sarcopenia, and carnitine is central to mitochondrial fatty acid metabolism. Therefore, for in vivo experiments, we constructed a high‐fat diet mouse model and treated model mice with carnitine. For in vitro experiments, we treated C2C12 myoblasts with palmitic acid and then with carnitine. Then RNA sequencing was used to analyse gene expression in different groups of mouse muscles and C2C12 cells. Enrichment analysis (Figure S5) revealed that DEGs between groups were highly enriched in ATP energy metabolism, myoblast development, oxidative stress and mitochondrial organisation. Considering the biological actions of carnitine in combination with the enrichment analysis results, we hypothesised that carnitine alters muscle cell fate by promoting fatty acid metabolism and thereby affecting mitochondrial morphology and function.

**FIGURE 5:**
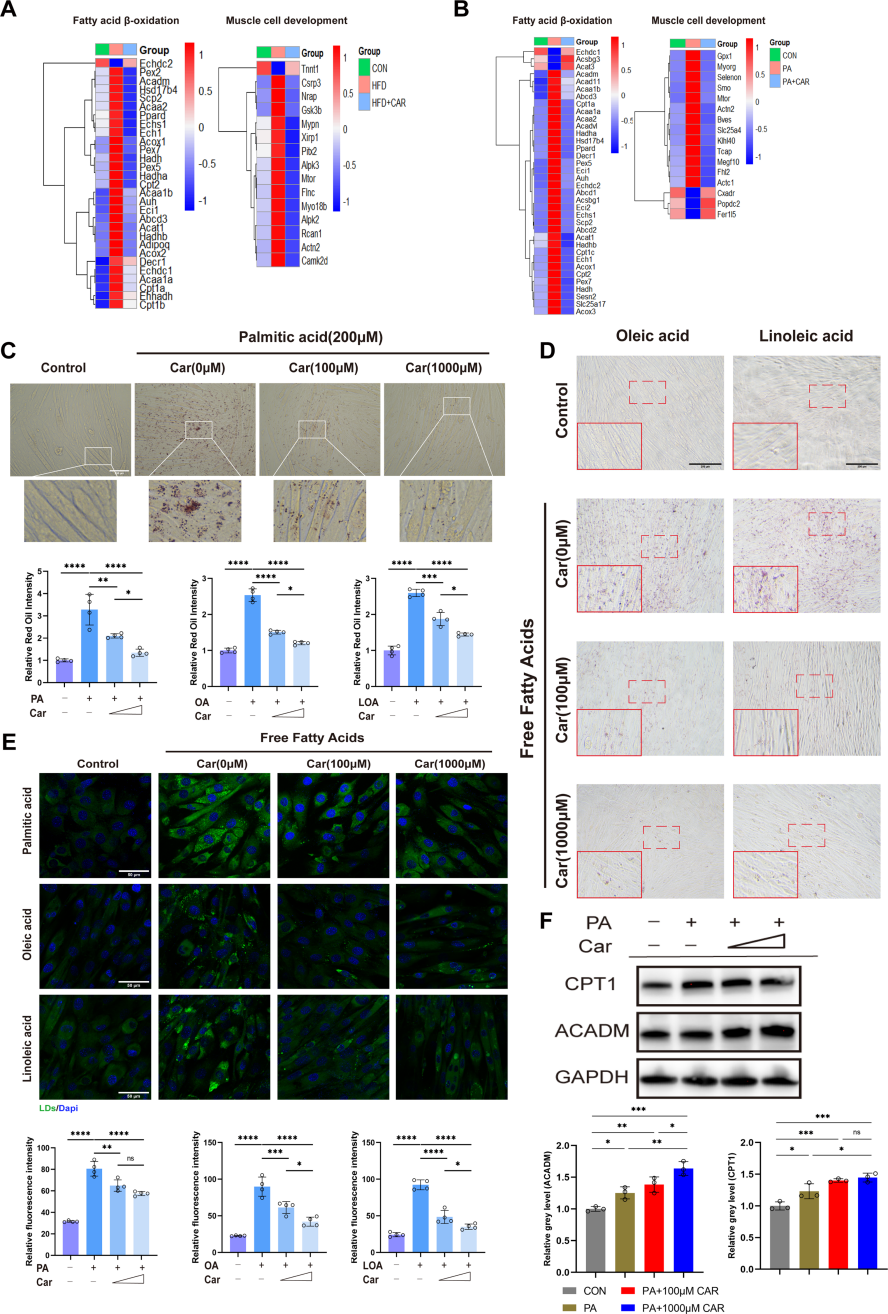
Carnitine reduces lipid accumulation caused by excess fatty acids. (A) Heatmap of the relative expression levels of *β*‐oxidation‐related genes and muscle differentiation‐related genes in the control (*n* = 3), high‐fat diet (*n* = 3) and carnitine treatment groups (*n* = 3) analysed via RNA‐Seq. (B) Heatmaps of the relative expression levels of *β*‐oxidation‐related genes and muscle differentiation‐related genes in the cells of the control (*n* = 3), palmitic acid‐treated (*n* = 3) and carnitine‐treated groups (*n* = 3) analysed via RNA‐Seq. (C and D) C2C12 myotube cells were treated with different free fatty acids (palmitic acid, oleic acid and linoleic acid) and different concentrations of carnitine for 24 h. Oil red O staining was performed to assess lipid accumulation. Relative quantitative analyses were performed. (E) C2C12 myotubular cells were treated with different free fatty acids (palmitic acid, oleic acid and linoleic acid) and different concentrations of carnitine for 24 h. Lipid droplet formation was assessed via fluorescence staining with BODIPY 493/503. Relative quantitative analysis was performed. (F) Western blot experiments were performed to assess the protein expression of CPT1 and ACADM in C2C12 myotubes treated with palmitic acid (200 μM) or different concentrations of carnitine (100 μM, 1000 μM). *p < 0.05, **p < 0.01, ***p < 0.001, ***p < 0.0001, means ± SDs.

First, gene expression profiles related to fatty acid beta‐oxidation as well as muscle cell development were obtained (Figure 5A,B). Compared with those in the respective control groups, gene expression was abnormal in the high‐fat diet‐fed mice and palmitic acid‐treated cells; however, carnitine treatment reversed this abnormal expression. Next, C2C12 myoblasts were treated with various free fatty acids, such as palmitic, oleic and linoleic acids. Oil red O staining revealed lipid accumulation in muscle cells, an effect that was significantly reduced by cotreatment with carnitine (Figure 5C,D). Bodipy 493/503 lipid droplet staining revealed similar results, with a significant increase in the number of lipid droplets after various fatty acid treatments and a significant decrease after carnitine cotreatment (Figure 5E). Western blot revealed that compared with those in the PA group, the levels of the *β*‐oxidation indicators CPT1 and ACADM in the carnitine cotreatment group were higher (Figure 5F). The above experimental results demonstrate that carnitine increases fatty acid mitochondrial *β*‐oxidation and reduces lipid accumulation.

### 3.6 Carnitine Reverses Lipid‐Induced Mitochondrial Damage

**FIGURE 6:**
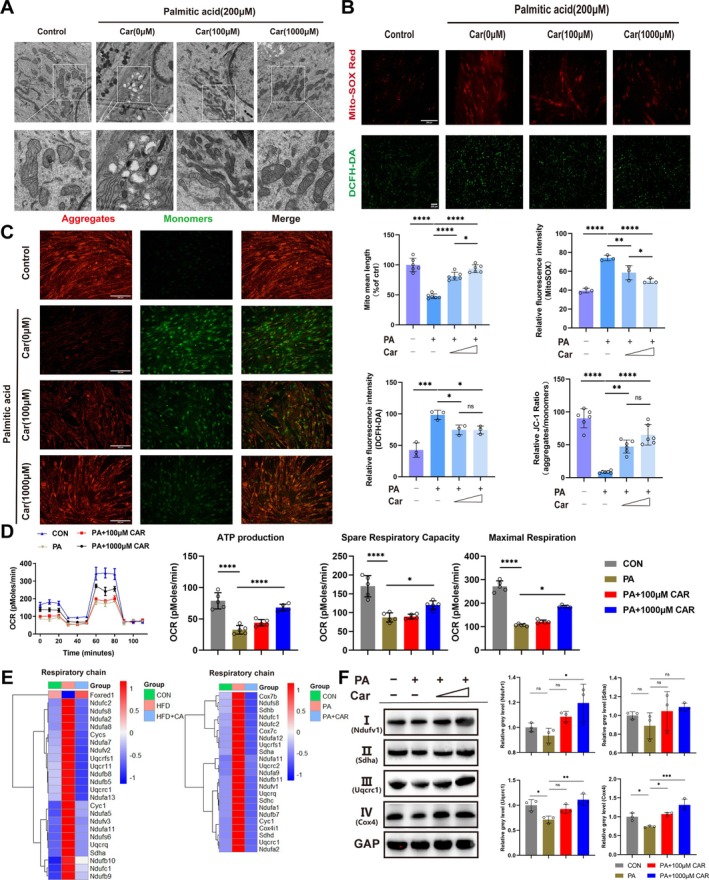
**Carnitine reverses lipid‐induced mitochondrial damage.** (A) C2C12 myotubes were treated with palmitic acid and different concentrations of carnitine and morphological changes in the mitochondria were assessed via transmission electron microscopy. The mitochondrial long axis diameter was measured. (B) C2C12 myotubes were treated with palmitic acid and different concentrations of carnitine; quantitative analysis of MitoSOX Red fluorescence (red) intensity (*n* = 3). Intramitochondrial ROS were detected with a MitoSOX Red probe and intracellular ROS were detected with a DCFH‐DA fluorescent probe; quantitative analysis of DCFH‐DA fluorescence (green) intensity (*n* = 3). (C) C2C12 myotubes were treated with palmitic acid and different concentrations of carnitine and JC‐1 staining was used to assess the mitochondrial membrane potential; quantitative analysis of red/green fluorescence intensity showing the ratio of aggregated JC‐1 to monomeric JC‐1 under different treatment conditions. (D) A Seahorse mitochondrial respiration assay was used to assess the effects of palmitic acid and different concentrations of carnitine on C2C12 myotube cells. Maximum respiration, spare capacity and ATP production were analysed. (E) Heatmap of relative expression levels of mitochondrial respiratory chain‐related genes in control (*n* = 3), high‐fat diet‐fed (*n* = 3) and carnitine‐treated mice (*n* = 3) analysed via RNA‐Seq; Heatmap of the relative expression levels of mitochondrial respiratory chain‐related genes in the cells of the control (*n* = 3), palmitate‐treated (*n* = 3) and carnitine‐treated groups (*n* = 3) analysed via RNA‐Seq. (F) Western blot experiments were performed to assess the protein expression of Ndufv1, Sdha, Uqcrc1 and Cox4 in C2C12 myotubes treated with palmitic acid (200 μM) or different concentrations of carnitine (100 μM and 1000 μM). Quantitative analysis of the grayscale ratios of protein bands for Ndufv1, Sdha, Uqcrc1 and Cox4 and for GAPDH (*n* = 3). *p < 0.05, **p < 0.01, ***p < 0.001, ****p < 0.0001, means ± SDs.

The changes in the gene profiles associated with oxidative stress and mitochondrial organisation were investigated (Figure S6). Similar to the previous results, compared with the respective control groups, high‐fat diet‐fed mice and palmitic acid‐treated cells presented altered gene expression, effects that were reversed by treatment with carnitine. Electron microscopy revealed an abnormal arrangement of mitochondrial cristae and vacuoles and a decreased mitochondrial density in palmitic acid‐treated cells, suggesting that the mitochondria were damaged; however, carnitine treatment restored the mitochondrial state (Figure 6A). A mitochondrial membrane potential assay revealed that the addition of palmitic acid caused a significant decrease in the mitochondrial membrane potential, suggesting impaired mitochondrial function, whereas carnitine treatment reversed the decrease in the mitochondrial membrane potential (Figure 6C). Mitochondria are the main generators of ROS in cells. MitoSOX experiments show that palmitic acid treatment significantly increased mitochondrial ROS levels. However, the addition of carnitine resulted in a dose‐dependent decrease in ROS levels in mitochondria (Figure 6B). Similarly, carnitine significantly reduced ROS levels in the cytoplasm, as detected with a DCFH‐DA fluorescent probe (Figure 6B).

On the basis of the changes in mitochondrial ultrastructure caused by excess lipids, we investigated the function of mitochondria in energy metabolism. Figure 6E shows the mitochondrial respiratory chain‐related gene expression profiles. Similar to previous results, excess lipids led to abnormalities and the incorporation of carnitine reversed these abnormalities. Ndufv1, Sdha, Uqcrc1 and Cox4 are essential subunit proteins in complex I, complex II, complex III and complex IV, respectively. Western blot analysis revealed that the addition of palmitic acid led to a decrease in the levels of these proteins, especially those of complex III (Uqcrc1) and complex IV (Cox4), whereas the addition of carnitine led to a dose‐dependent increase in protein expression levels (Figure 6F). Seahorse technology revealed that the basal oxygen consumption rate of the cells in the palmitic acid group was lower than that in the control group, indicating a decrease in oxidative phosphorylation activity. However, carnitine treatment resulted in a dose‐dependent increase in the basal oxygen consumption rate. In addition, the maximum respiration rate, mitochondrial ATP production rate and spare respiratory capacity of the cells in the palmitic acid group were lower than those in the control group and carnitine treatment caused a dose‐dependent increase in the above indices (Figure 6D). Taken together, these findings suggest that carnitine reverses mitochondrial structure and function disruption caused by excess lipids and maintains mitochondrial homeostasis.

### 3.7 Carnitine Administration Ameliorates Muscle Atrophy in Conditions of High‐Fat Diet or OCTN2 Deficiency

Next, to explore whether carnitine could rescue the muscle atrophy phenotype caused by OCTN2 deficiency, we injected mice with shNC versus sh‐OCTN2 adenovirus and explored the effect of carnitine on it (Figure S7A). In the sh‐OCTN2 group, carnitine was able to reverse the grip strength loss phenotype in sh‐OCTN2 mice (1.527 ± 0.035 N vs. 1.678 ± 0.084 N, *p* < 0.05; Figure S7B), restoring myofiber cross‐sectional area (1.21*10^−3^ ± 5.8*10^−5^ mm^2^ vs. 1.381*10^−3^ ± 7.89*10^−5^ mm^2^, *p* < 0.05) and myofiber number (Figure S7C,D). However, in the shNC group, carnitine treatment did not significantly affect grip strength, muscle fibre cross‐sectional area and muscle fibre number (Figure S7B–D). There was no significant change in body weight in all four groups of mice (Figure S7B). The above results suggest that carnitine can partially rescue muscle atrophy caused by OCTN2 deficiency.

**FIGURE 7:**
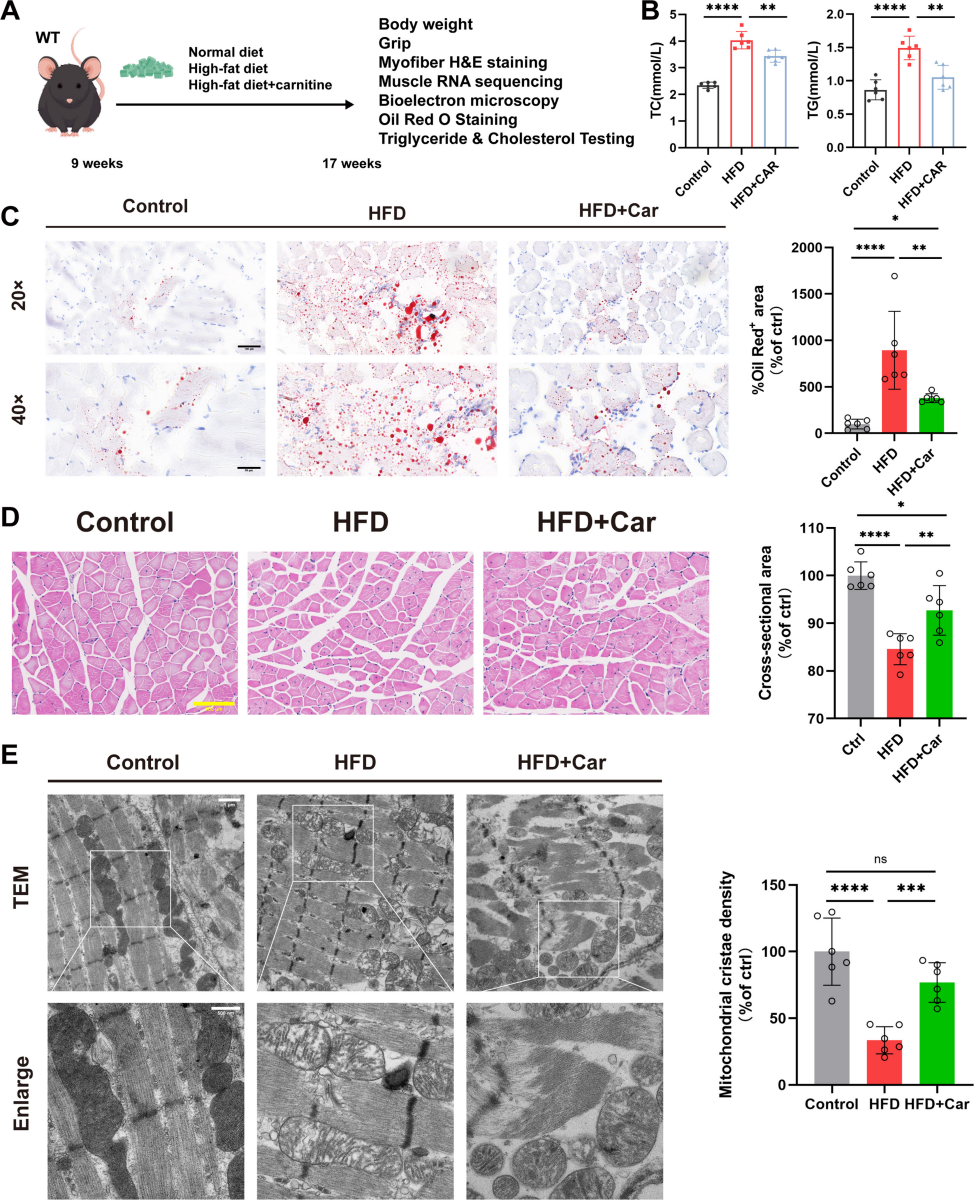
Carnitine administration ameliorates high‐fat diet‐induced muscle atrophy. (A) Experimental design model. (B) Blood triglyceride and cholesterol levels. (C) Representative images of oil red O‐stained mouse gastrocnemius muscle. The oil red O‐positive area/total area was quantified via ImageJ software. (D) H&E staining of the mouse gastrocnemius muscle and measurement of the cross‐sectional area of the sections. (E) Transmission electron microscopy of mouse skeletal muscle mitochondrial morphology and quantitative statistics of mitochondrial crista density. *p < 0.05, **p < 0.01, ***p < 0.001, ****p < 0.0001, means ± SDs.

Mice that were fed a high‐fat diet and treated with carnitine were analysed (Figure 7A). Carnitine reduced blood cholesterol (4.035 ± 0.328 mmol/L vs. 3.432 ± 0.223 mmol/L, *p* < 0.01) and triglyceride (1.492 ± 0.177 mmol/L vs. 1.052 ± 0.179 mmol/L, *p* < 0.01) levels in high‐fat diet‐fed mice (Figure 7B). Skeletal muscle cryosections stained with oil red O revealed that the high‐fat diet aggravated fat infiltration into skeletal muscle in the mice, an effect that was attenuated by carnitine treatment (Figure 7C). H&E staining analysis revealed that muscle fibres were smaller in high‐fat diet‐fed mice than in control mice and that carnitine treatment reversed that phenomenon (Figure 7D). Electron microscopy revealed that the number and density of mitochondrial cristae were reduced in the skeletal muscle of the high‐fat diet group, suggesting that the mitochondria were damaged. However, carnitine treatment restored the mitochondrial state (Figure 7E). This finding is consistent with the findings of our cellular experiments. These results show that carnitine prevents muscle atrophy by reducing lipid accumulation and maintaining mitochondrial homeostasis in skeletal muscle in vivo, providing a new strategy for treating sarcopenia.

## 4 Discussion

In this study, we found that OCTN2 and carnitine were strongly associated with the development of sarcopenia. Serum carnitine levels decreased in patients with sarcopenia. Both OCTN2 and carnitine levels decreased in sarcopenia animal models and in aged mice. OCTN2 deficiency resulted in decreased carnitine content and consequent muscle atrophy, as well as a reduced response to exercise‐induced muscle strengthening. A mechanistic study revealed that mechanical force enhanced OCTN2 transcription via YAP/TEAD4, which regulated carnitine homeostasis. The YAP agonist XMU alleviated muscle atrophy symptoms in mice. Further experiments suggested that carnitine maintained normal cellular metabolism by reducing lipid accumulation to maintain mitochondrial homeostasis.

Previous studies have indicated that carnitine and OCTN2 levels decrease with age during the aging process [29, 30]. Sarcopenia often occurs during the aging process. Carnitine is critical in skeletal muscle metabolism. L‐carnitine prevents apoptosis in skeletal muscle cells and plays a role in the treatment of myopathy associated with congestive heart failure [31]. L‐carnitine supplementation has been shown to enhance muscle function and athletic tolerance [32] and reduce fatigue [33]. However, all of the above studies focused on phenotypic studies of exogenous carnitine supplementation therapy; endogenous carnitine has not been studied in terms of the pathological mechanisms of sarcopenia. The therapeutic value of endogenous carnitine for sarcopenia has also not been studied. Our study focused on the carnitine transporter channel protein OCTN2 and partially complements studies on the pathological mechanisms of endogenous carnitine in sarcopenia. Mutations in the SLC22A5 gene encoding OCTN2 cause primary carnitine deficiency. The phenotypes of this disease include decreased carnitine levels and skeletal muscle disease [10]. Does this suggest a correlation between OCTN2 deficiency and skeletal muscle disease? Our results suggest that decreased carnitine content due to OCTN2 deficiency is an important factor in human sarcopenia. Carnitine and OCTN2 levels are reduced in human serum as well as in the muscles of several muscle atrophy model mice. To explore the relationship between OCTN2 and muscle atrophy, we first constructed a C2C12 cell line stably transformed with sh‐OCTN2 via lentivirus, and the experimental results revealed that myotube formation was impaired in C2C12 cells with OCTN2 knockdown. Further electron microscopy experiments revealed that the mitochondrial structure was damaged. We subsequently constructed an AAV‐shOCTN2 adenovirus and injected it intramuscularly into the hind limbs of mice. The results showed that the knockdown of OCTN2 caused a muscle atrophy phenotype in the mice, which may have been due to a decrease in the carnitine concentration. Previous studies have shown that patients with hepatocellular carcinoma treated with sorafenib have decreased serum carnitine levels and sarcopenia, effects that may be attributed to the inhibition of OCTN2 by sorafenib [34] and our results confirmed that decreased carnitine content due to the knockdown of OCTN2 results in muscle wasting.

As reported in previous studies, exercise can influence the metabolic state of carnitine [26] and carnitine levels are biomarkers of sarcopenia and nutritional status [35]. Muscles, as important parts of the locomotor system, are closely related to exercise [20, 36]. Exercise has been shown to enhance muscle function. However, it remains unclear whether OCTN2‐mediated alterations in carnitine content are modulated by mechanical forces. We found that muscle OCTN2 expression was elevated in swimming‐exercised mice and that OCTN2 content was decreased in aged mice, an effect that may be related to the lack of exercise in aged and frail individuals. Mechanical stimulation is a key physical factor in the alteration of the cellular environment. To date, we have identified multiple signalling pathways that are regulated by mechanical forces during mechanosignalling and that have an impact on skeletal muscle. For example, the Hippo–YAP signalling pathway consists of a series of conserved kinases and YAP activation promotes an increase in muscle fibre volume [21]. PIEZO1 is a cell membrane protein that directly senses extracellular mechanical stress. The knockdown of PIEZO1 reduces the concentration of calcium ions in skeletal muscle, which weakens muscle contraction and causes muscle atrophy [22]. Wnt/*β*‐catenin signalling promotes acetylcholine receptor aggregation at the neuromuscular junction in response to mechanical force stimulation of muscle contraction [23]. In addition, mechanical strain activates Rho/ROCK signalling and regulates the fate of myoblast differentiation by modulating the downstream MAPK pathway [24]. YAP and TAZ, as critical factors in the Hippo signalling pathway, are mechanosensitive transcriptional modulators. They can be regulated by various mechanical forces, including shear stress, cell shape and extracellular matrix rigidity, thereby affecting cell‐specific transcriptional programmes [37]. Additionally, mechanical forces can directly drive the nuclear translocation of YAP by reducing the mechanical restriction of molecular transport at nuclear pores of the cell nucleus [38]. In the nucleus, YAP/TAZ can interact with transcription factors such as Tead1–4, a member of the TEA structural domain family, to regulate gene expression and promote growth processes [39]. In this study, the regulation of OCTN2 by YAP was most pronounced. YAP bound to TEAD4 to increase OCTN2 transcription. Moreover, mechanical stimulation strengthened the binding of YAP and TEAD4 and the transcription of OCTN2. To confirm whether exercise prevents muscle atrophy via OCTN2/carnitine, we subjected sh‐OCTN2‐injected mice to swimming exercise. Exercise increased the muscle fibre area and grip strength in control mice, but the increase in muscle fibre area and grip strength was not significant in sh‐OCTN2 mice. However, the injection of the YAP agonist XMU alleviated DEX‐induced muscle atrophy in mice, a finding that is consistent with previous results.

Next, we investigated how carnitine in cells affects the fate of muscle cells. Previous studies have reported that impaired lipid metabolism is one of the causes of the progression of sarcopenia and that excessive lipid infiltration affects normal cellular metabolism, increases reactive oxygen species production, leads to lipotoxicity and insulin resistance and induces the production of inflammatory factors that ultimately lead to obesity‐related sarcopenia [13]. Interestingly, the targeted destruction of YAP in adult skeletal muscle also leads to the disruption of fatty acid oxidation and thus lipid toxicity [21]. This finding is similar to our results. The major function of carnitine is to transport long‐chain fatty acids, which are not permeable to mitochondria, into the mitochondria to supply beta‐oxidation energy. In our study, in a cellular model of free fatty acid treatment, large lipid droplets formed and the function and morphology of the mitochondria were impaired; however, carnitine treatment reduced the accumulation of lipid droplets and restored the function and morphology of the mitochondria. A high‐fat diet also induced lipid accumulation in muscle cells, mitochondrial damage and a muscle atrophic phenotype, whereas carnitine intake reduced the accumulation of lipids in muscle, restored mitochondrial morphology and ameliorated muscle atrophy. These data demonstrate the ability of carnitine to ameliorate muscle atrophy by promoting fatty acid metabolism, reducing lipid accumulation and maintaining mitochondrial homeostasis.

**FIGURE 8:**
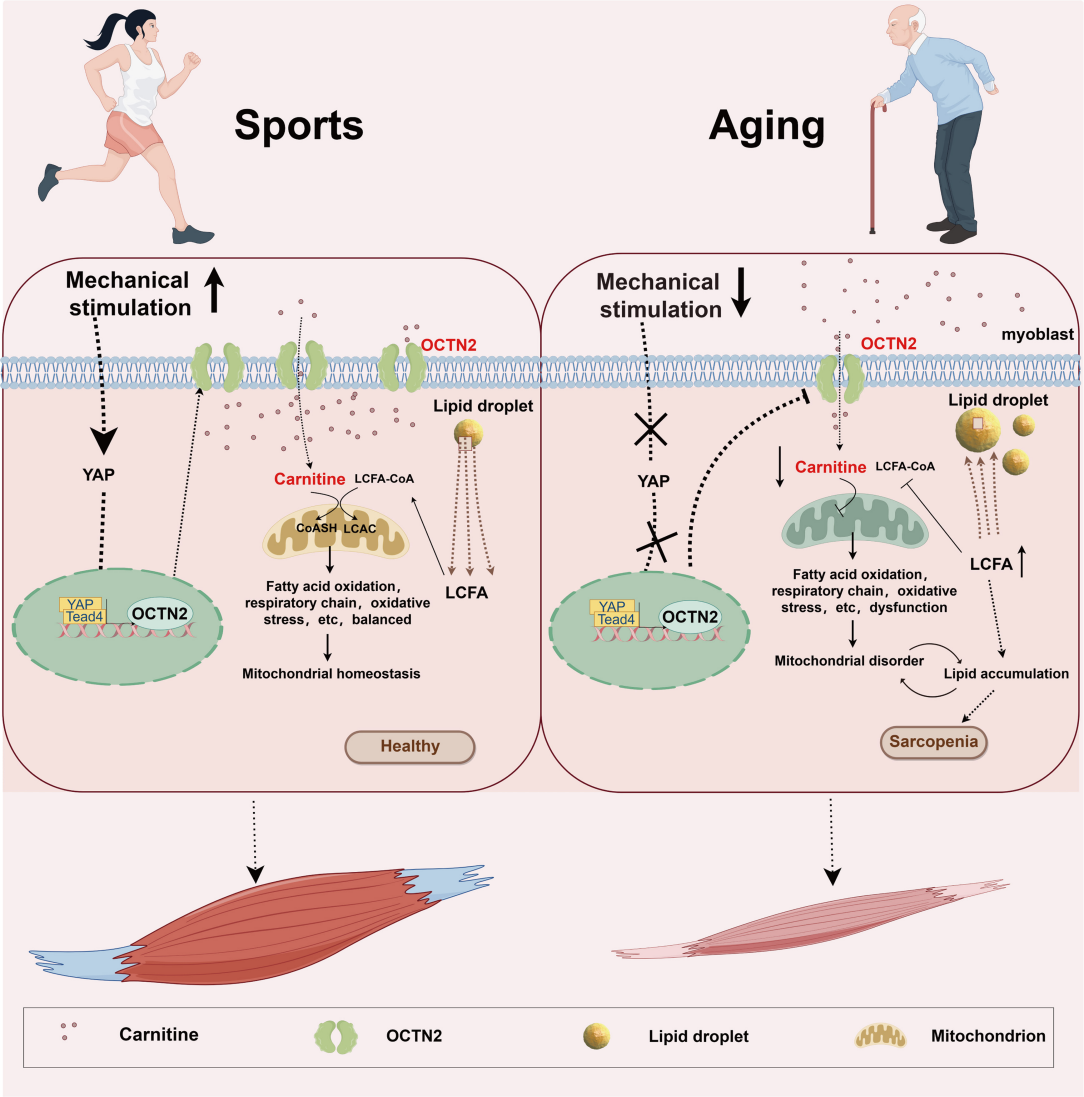
Graphic summary of this study. Under physiological conditions, stress stimulation promotes the transcription and expression of the specific carnitine transport channel protein OCTN2 through the YAP/TEAD4 pathway. Muscle cells take up sufficient amounts of carnitine into the cytosol via OCTN2. Carnitine in the cytoplasm selectively binds fatty acids and then enters the mitochondria for lipid metabolism to provide energy and maintain the physiological function of muscle cells. Under aging conditions, impaired lipid metabolism and lipid accumulation in muscle cells, as well as a decrease in OCTN2 expression due to reduced mechanical stimulation, lead to a decrease in carnitine uptake by muscle cells. The combined effects of these two factors lead to dysfunctions in mitochondrial *β*‐oxidation and the electron respiratory chain and to oxidative stress in muscle cells, which in turn aggravate lipid accumulation, generating a vicious cycle. Lipotoxicity and mitochondrial dysfunction caused by lipid accumulation in muscle cells accelerate muscle cell aging and ultimately lead to the development of sarcopenia.

In conclusion, our study demonstrated that OCTN2 deficiency affects muscle carnitine content, which influences skeletal muscle fate by regulating fatty acid metabolism and maintaining mitochondrial homeostasis. We also demonstrated that mechanical forces can regulate OCTN2 expression through YAP/TEAD4 (Figure 8). In fact, bedridden and aging individuals who are physically inactive tend to experience a reduction in the diameter of skeletal muscle fibres, resulting in sarcopenia [40, 41]. The mechanisms involved may be complex. Our findings at least demonstrate that OCTN2‐mediated (regulated by mechanical forces) alterations in carnitine content can have some effect on normal cellular metabolism in muscle and that this effect can lead to sarcopenia. Our findings suggest that carnitine can couple mechanical stimulation and fatty acid metabolism to influence muscle cell fate; mechanical interventions as well as the targeting of OCTN2 to increase the carnitine content may be therapeutic approaches for preventing sarcopenia.

## Conflicts of Interest

The authors declare no conflicts of interest.

## Supporting information


**Figure S1:** (A) Clinical characteristics of the patients. (B and C) H&E staining of mouse gastrocnemius muscle and measurement of the cross‐sectional area of the sections. (D) The grip strength and body weight of DEX‐treated mice were measured over a two‐week period.
**Figure S2:** (A) Western blot analysis of myotube OCTN2 protein expression at days 0, 1, 3 and 6 of differentiation. The intensity of the OCTN band relative to that of the GAPDH band was quantified via ImageJ software (*n* = 3). (B) mRNA expression of OCTN2 was assessed by RT–qPCR at different stages of C2C12 differentiation. (C)Western blot analysis of the protein levels in the expression of shOCTN2‐Injected Mice and shNC‐Injected Mice. (D) Fatty acid oxidising activity in muscle of shOCTN2‐injected mice and shNC‐injected mice.
**Figure S3:** (A) Experimental design model. (B) Western blotting was used to analyse the expression levels of phospho‐YAP, YAP and OCTN2 in C2C12 myotubes treated with tension force for 6 h or not and then cultured for 18 h. RT–qPCR analysis of the expression levels of YAP and OCTN2 in C2C12 myotubes treated with tension force for 6 h or not and then cultured for 18 h. (C) RT–qPCR analysis of OCTN2 mRNA expression under tension stimulation by Yap silencing.
**Figure S4:** (A) Schematic diagram of modelling of mice injected with sh‐OCTN2 adenovirus for swimming exercise. (B) Schematic diagram of modelling of XMU‐treated DEX‐induced myasthenia gravis mice.
**Figure S5:** (A) GO enrichment analysis of the control (*n* = 3) and high‐fat diet groups (*n* = 3). (B) GO enrichment analysis of the high‐fat diet (*n* = 3) and carnitine‐treated groups (*n* = 3). (C) KEGG enrichment analysis of palmitic acid (*n* = 3) and carnitine‐treated groups (*n* = 3). (D) GO enrichment analysis of the palmitic acid (*n* = 3) and carnitine‐treated groups (*n* = 3).
**Figure S6:** Heatmap analysis of RNA sequencing results in mice and cells. (A) Heatmap of the relative expression levels of oxidative stress‐related genes and mitochondrial tissue‐related genes in the control (*n* = 3), high‐fat diet (*n* = 3) and carnitine‐treated groups (*n* = 3), as analysed via RNA‐Seq. (B) Heatmaps of the relative expression levels of oxidative stress‐related genes and mitochondrial tissue‐related genes in control (*n* = 3), palmitic acid‐treated (*n* = 3) and carnitine‐treated (*n* = 3) cells analysed via RNA‐Seq.
**Figure S7:** Carnitine supplementation partially rescues the muscle atrophy phenotype in OCTN2 deficiency conditions. (A) Experimental design model. (B) Grip strength and body weight of the mice. (C) H&E staining of the mouse gastrocnemius muscle. (D) Myofiber cross‐sectional area statistics and myofiber number statistics.
**Table S1:** Association between serum carnitine and sarcopenia.
**Table S2:** Reagents and antibodies.
**Table S3:** Sequence of the primers used for qPCR.
**Table S4:** Sequence of the primers used for Chromatin immunoprecipitation (ChIP) assay.
**Table S5:** Goutallier classification system.
**Table S6:** Details of the equipment used for MRI and DXA.
