# Supplementary material for: Mechanical Stimulation Induces Yap Mediated OCTN2 Transcription to Enhance Carnitine Metabolism in Sarcopenia
Source: J Cachexia Sarcopenia Muscle. 2025 Sep 17;16(5):e70052. doi: 10.1002/jcsm.70052 (PMC12441309; doi:10.1002/jcsm.70052)

Supplementary Materials for

**Mechanical stimulation induces Yap mediated OCTN2 transcription to enhance carnitine metabolism in sarcopenia**

Yahong Lu1,† , Yu Bai1,†, Weiqing Li1,†, Zhiguo Zhou1, Heihuan Lai1, Xingyu Hu1, Tao Yang1,2, Chendi Wang1, Yitao Chen1, Keping Gan1, Kechi Li1, Haiwei Ma1,*, Lin Shen1,*, Dengwei He1,*

**This supplemental material file includes:**

Materials and Methods

Figs. S1 to S7

Tables S1 to S6

uncropped original gel images

**Materials and methods**

**Mouse exercise protocol**

Mice were trained to swim in a container (54 cm*40 cm*28 cm) with a depth of 20 cm; the water temperature was 35~37°C. The first week of swimming was the adaptation period, starting with 10 min, after which the training time was increased by 10 min per day until the training time reached 50 min, after which the training time was maintained at 50 min per day. After swimming, the mice were wiped with a towel and returned to their original cages. Swimming training was performed 5 days per week for 8 weeks.

**Muscle atrophy mouse model**

Fourteen 9-week-old C57BL/6J mice were randomly assigned to three groups: mice in the control group was given PEG400 solution (10 mL/kg body weight, 30% dissolved in 0.9% saline); mice in the DEX group were intraperitoneally injected with DEX solution (25 mg/kg dissolved in PEG400 solution once a day for 14 days); and mice in the DEX+XMU group were administered a DEX solution (25 mg/kg dissolved in PEG400 solution, administered as above) and an XMU solution (1 mg/kg, dissolved in corn oil, every other day for 14 days) intraperitoneally. Grip strength and body weight were monitored during this period. The mice were sacrificed 24 h after the last injection, and the gastrocnemius, tibialis anterior, piriformis, and quadriceps muscles were collected for subsequent analyses.

**High-fat diet mouse model**

Nine-week-old C57BL/6J mice were assigned to three groups, and after two weeks of acclimatization, mice in the control group were fed a regular chow (10% kcal of fat, D12450J, Research Diets), and mice in the other groups were fed a high-fat diet (60% kcal of fat, D12492, Research Diets). Mice in the HFD+CAR group was given 100 mg/kg/day L-carnitine through their drinking water (MCE; Shanghai, China). All the mice were fed for 8 weeks. Before sacrifice, the mice were fasted for 6 h, and tissues were collected according to the appropriate preparation method for each experiment.

**Adeno-associated virus administration model**

C57BL/6 mice in the same cage were randomly assigned to the sh-NC group or the sh-OCTN2 group. Five sites were selected for adenovirus injection into the tibialis anterior, gastrocnemius, and quadriceps muscles of mice, and each site was injected with 10 µl of HBAAV2/9-m-Slc22a5 shRNA1-EGFP (2.6*10^12^vg/mL) or HBAAV2/9-EGFP NC (1.5*10^12^vg/mL). Western blot analysis of OCTN2 protein expression in mouse muscle was performed to verify the effect of adenovirus knockdown by sh-OCTN2. The mice were weighed before sacrifice, and grip strength was tested three times with a mouse grip meter. The gastrocnemius, tibialis anterior, piriformis, and quadriceps muscles were collected after mouse sacrifice for subsequent analyses.

**Cell culture and in vitro treatments**

C2C12 cells (a mouse myoblast cell line) were cultured in DMEM + 10% FBS at 37°C in a 5% CO2 incubator. When the cells reached a confluence of 90%, the medium was changed to DMEM containing 2% horse serum, and the cells were cultured for 5–7 days to form multinucleated myotubes. The medium was changed every 24 h.

For mechanical stimulation, cells were seeded at a confluence of 90% into type I collagen-coated elastic cell culture plates, and after attachment, a CELL TALK instrument (Hangzhou Surface & Force Technology, Hangzhou, China) was used to stimulate the cells with stretching at 5% tension rate 1 cycle per second for 6 hours.

**Reagents and antibodies**

Information on reagents and antibodies is detailed in Table S2

**RNA isolation and qPCR**

Cells were lysed with TRIzol (Vazyme, Nanjing, China) for RNA extraction. cDNA was reverse transcribed using a kit following the manufacturer's instructions. Gene expression was quantified using SYBR Green Master Mix (Yeason) and an ABI 7500 instrument (Thermo Fisher Scientific). The sequences of primers used are detailed in Table S3.

**Transmission electron microscopy**

After treatment, cells were seeded in six-well plates, fixed with 2.5% glutaraldehyde and washed with PBS. Then, they were fixed in osmium acid with potassium ferricyanide for 30 min in the dark, incubated in 1% TCH for 30 min, fixed in 2% osmium acid for 30 min, and fixed in 2% hydrogen peroxide acetate for 1 h in a 37°C oven. Alcohol gradient dehydration, resin infiltration, embedding and polymerization with preprepared resin columns, ultrathin sectioning and postfixation staining were performed. The samples were observed using a transmission electron microscope.

**Metabolomics analysis**

Targeted metabolomics was performed by Health Bank Medical (Hangzhou, China). For sample preparation, accurately weighed tissue samples were placed into grinding tubes. At a ratio of 1:30 μL, the corresponding volume of isopropanol (containing 10 mM ammonium acetate) was added to the tissue extract and grinding beads, and the tissue samples were homogenized at 70 Hz × 180 s in an electric homogenizer (Jingxin Technology, Shanghai, China). Tissue homogenate was obtained. A total of 90 μL of tissue homogenate supernatant was mixed with 25 μL of internal standard mixture and vortexed at 1500 rpm × 5 min, followed by centrifugation at 5300 rpm × 20 min. The supernatant was transferred to a 96-well plate (Thermo Scientific, USA) for targeted lipid testing.

For sample testing, large-scale deep-targeted quantitative metabolomics analysis of mouse muscle tissue was performed via UPLC‒MS/MS. LC‒MS/MS quantification of targeted lipids was performed using an AB SCIEX Triple Quad™ 4500MD liquid chromatography tandem triple quadrupole mass spectrometry detection system (SCIEX, Singapore), with data acquisition in selective reaction detection mode (SRM). LC‒MS/MS quantification of acylcarnitines was performed using a Shimadzu LC‒20ADXR (Shimadzu, Japan)-AB SCIEX 5500 plus tandem triple quadrupole mass spectrometer (SCIEX, Singapore), and data were collected in selected reaction detection mode SRM.

Mass spectrometry data were processed via AB SCIEX OS-MQ software (SCIEX, Singapore). The stable isotope lipid internal standard single point correction method was used for quantification. Multivariate statistical analyses were performed using SIMCA software (Sartorius AG Umetrics, Göttingen, Germany), and t tests with multiple test correction were performed using RStudio. Metabolic pathway analyses were performed using the online website MetaboAnalyst (<https://www.metaboanalyst.ca/>). All metabolomics data are available in the National Genomics Data Center (NGDC) database under the sequence number OMIX009923.

**RNA sequencing**

RNA sequencing was performed by Shanghai Biochip (Shanghai, China). Adherent cells were lysed with sufficient TRIzol to extract RNA; for animal tissues, the target tissues were rapidly isolated and snap-frozen in liquid nitrogen, followed by RNA extraction. Briefly, total RNA extraction from the samples was performed using RNeasy (Qiagen) RNA extraction kits following the standard procedure provided by the manufacturer. The resulting RNA was subjected to electrophoretic QC with an Agilent 4200 TapeStation (Cat# G2991A, Agilent) and quantitative QC with a Qubit 3.0 fluorometer (Cat# Q33216, Thermo Fisher, CA, USA)/NanoDrop One (ND-ONE, Thermo Fisher Scientific) for quantitative QC. RNAs that passed quality control were used for subsequent library construction. The libraries were uploaded via the Illumina sequencing platform. Bioinformatics analysis of the generated data was performed using R studio. All sequencing data are available in the Gene Expression Omnibus (GEO) database under the sequence number GSE294869 and GSE295020.

**Chromatin immunoprecipitation assay**

We searched the promoter region 2000 bp upstream of the OCTN2 gene and used the TFDB (https://guolab.wchscu.cn/AnimalTFDB#!/) database to predict possible transcription factor-binding sites. The binding of TEAD4 to six OCTN2 promoter region binding sites was determined via a ChIP assay. Briefly, cells were crosslinked with 1% formaldehyde, digested, and sonicated to extract chromatin. The chromatin was then immunoprecipitated with an anti-TEAD4 antibody, an anti-rabbit IgG antibody (negative control), and an anti-H3 antibody (positive control), with 2% of the sample used as the input group. DNA was purified from the immunoprecipitated chromatin to be used as a PCR template, and primers were specifically designed to amplify fragments from each OCTN2 binding site. The primer sequences are listed in Table S4.

**Luciferase experiment**

The experiments were performed using a Dual-Luciferase Reporter Assay Kit (Vazyme, Nanjing, China). C2C12 cells were seeded and transfected with pGL3-Basic, pGL3-OCTN2-Luc, and OCTN2 promoter sequence plasmids with mutations at the corresponding sites. Forty-eight hours later, the culture supernatants were removed, and the cells were prepared for firefly luciferase and Renilla luciferase assays.

**Figure S1**


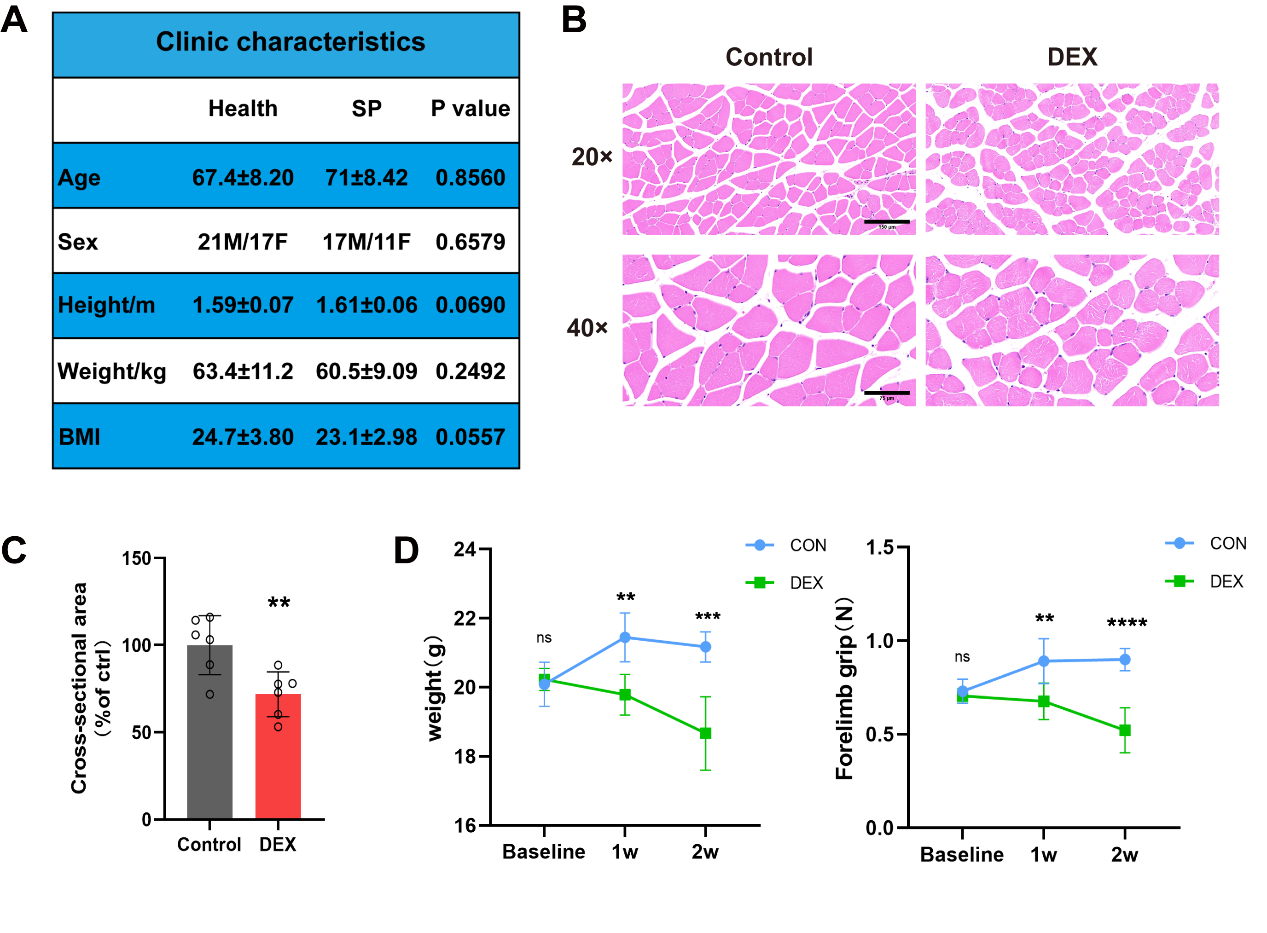


(A) Clinical characteristics of the patients.

(B and C) H&E staining of mouse gastrocnemius muscle and measurement of the cross-sectional area of the sections.

(D) The grip strength and body weight of DEX-treated mice were measured over a two-week period.

**Figure S2**
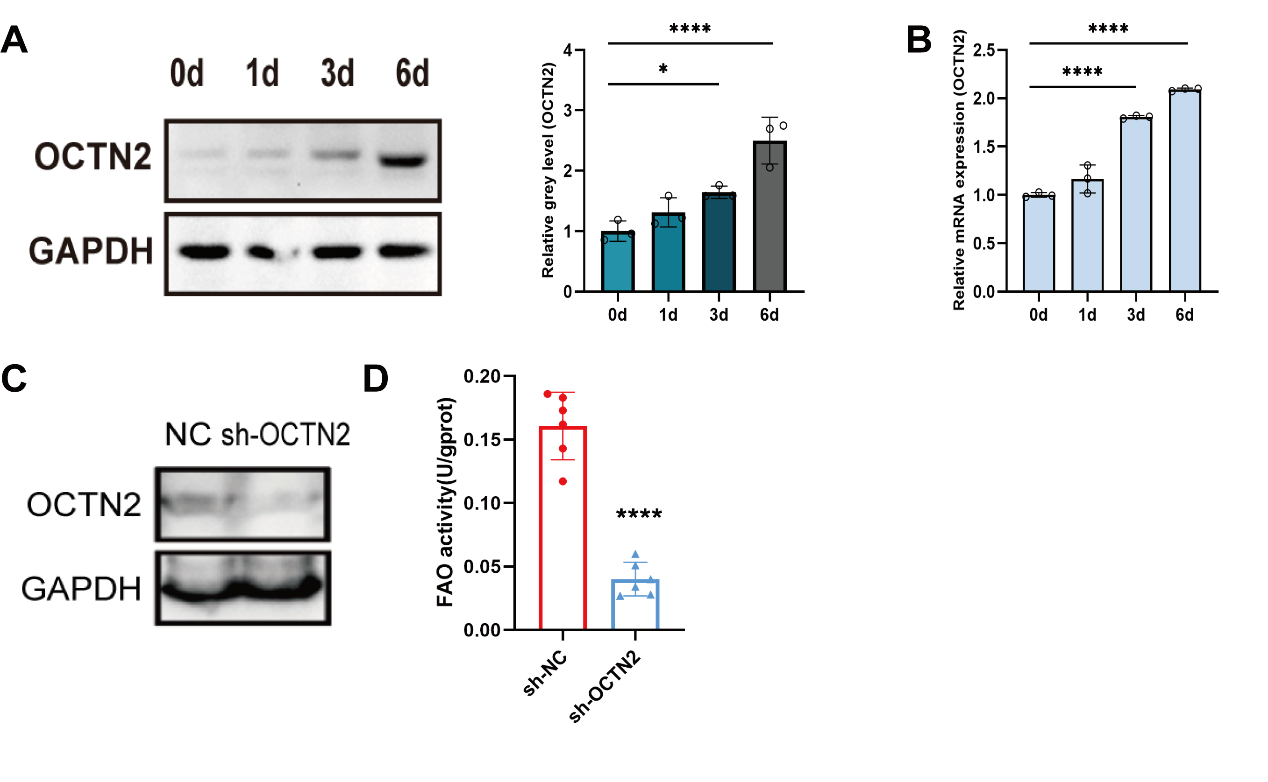


(A) Western blot analysis of myotube OCTN2 protein expression at days 0, 1, 3, and 6 of differentiation. The intensity of the OCTN band relative to that of the GAPDH band was quantified via ImageJ software (n=3).

(B) mRNA expression of OCTN2 was assessed by RT‒qPCR at different stages of C2C12 differentiation.

(C)Western blot analysis of the protein levels in the expression of shOCTN2-Injected Mice and shNC-Injected Mice.

(D) Fatty acid oxidizing activity in muscle of shOCTN2-injected mice and shNC-injected mice.

**Figure S3**


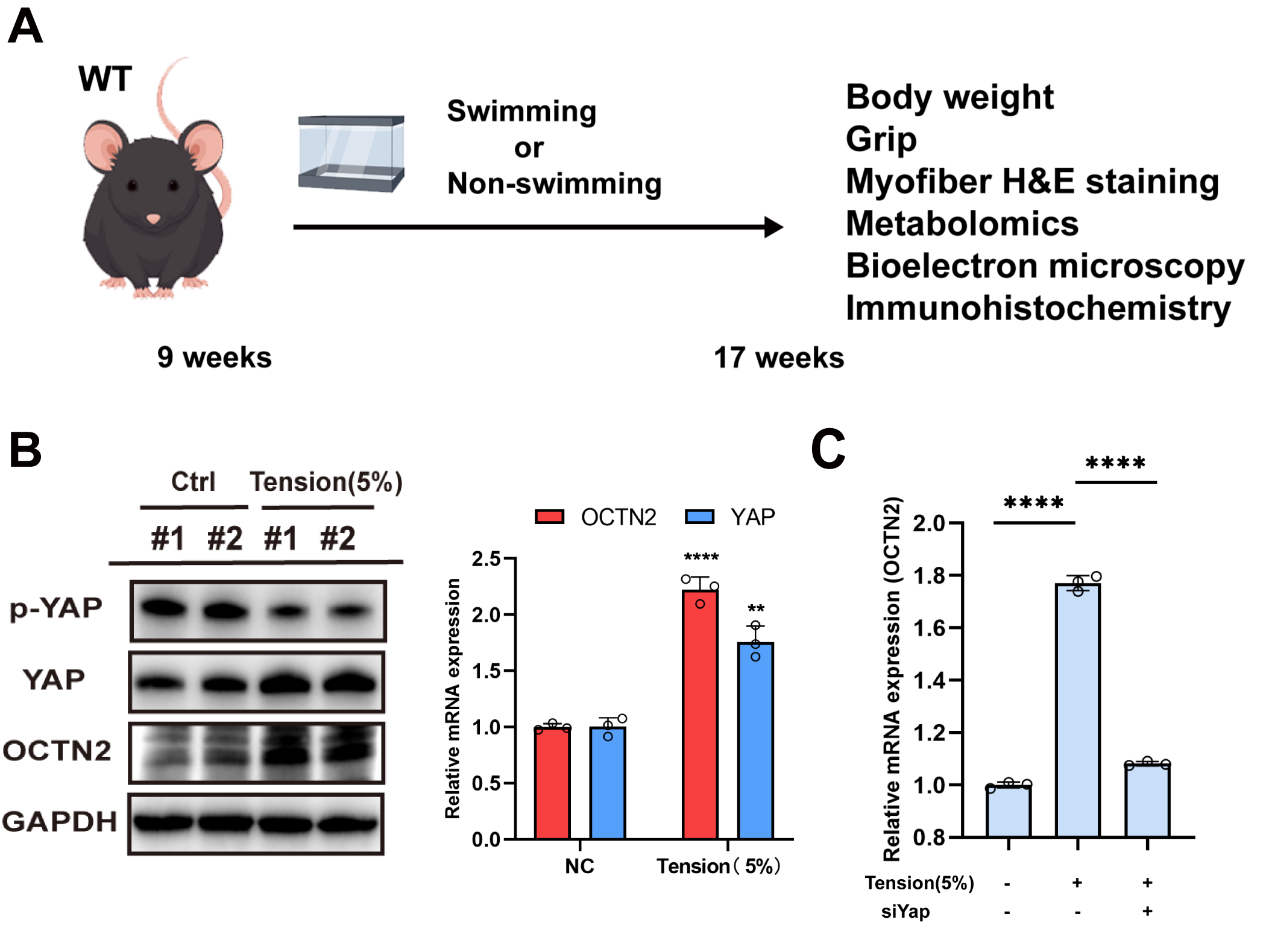


(A) Experimental design model.

(B) Western blotting was used to analyze the expression levels of phospho-YAP, YAP and OCTN2 in C2C12 myotubes treated with tension force for 6 h or not and then cultured for 18 h. RT‒qPCR analysis of the expression levels of YAP and OCTN2 in C2C12 myotubes treated with tension force for 6 h or not and then cultured for 18 h.

(C) RT‒qPCR analysis of OCTN2 mRNA expression under tension stimulation by Yap silencing.

**Figure S4**


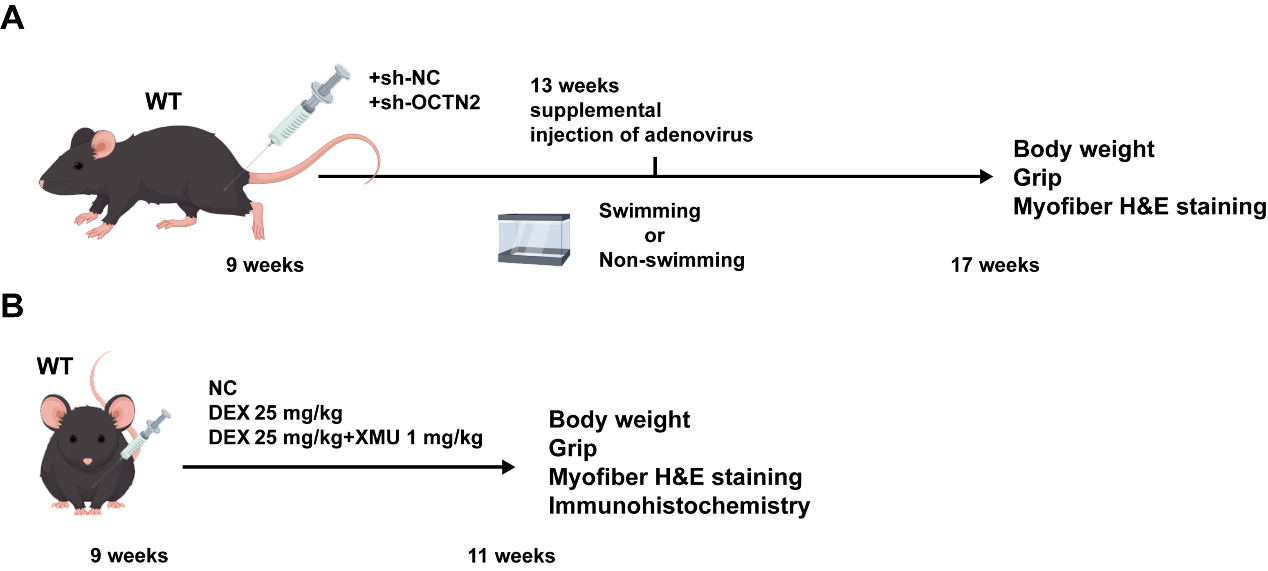


(A) Schematic diagram of modeling of mice injected with sh-OCTN2 adenovirus for swimming exercise..

(B) Schematic diagram of modeling of XMU-treated DEX-induced myasthenia gravis mice.

**Figure S5 RNA-Seq results in mice muscle**


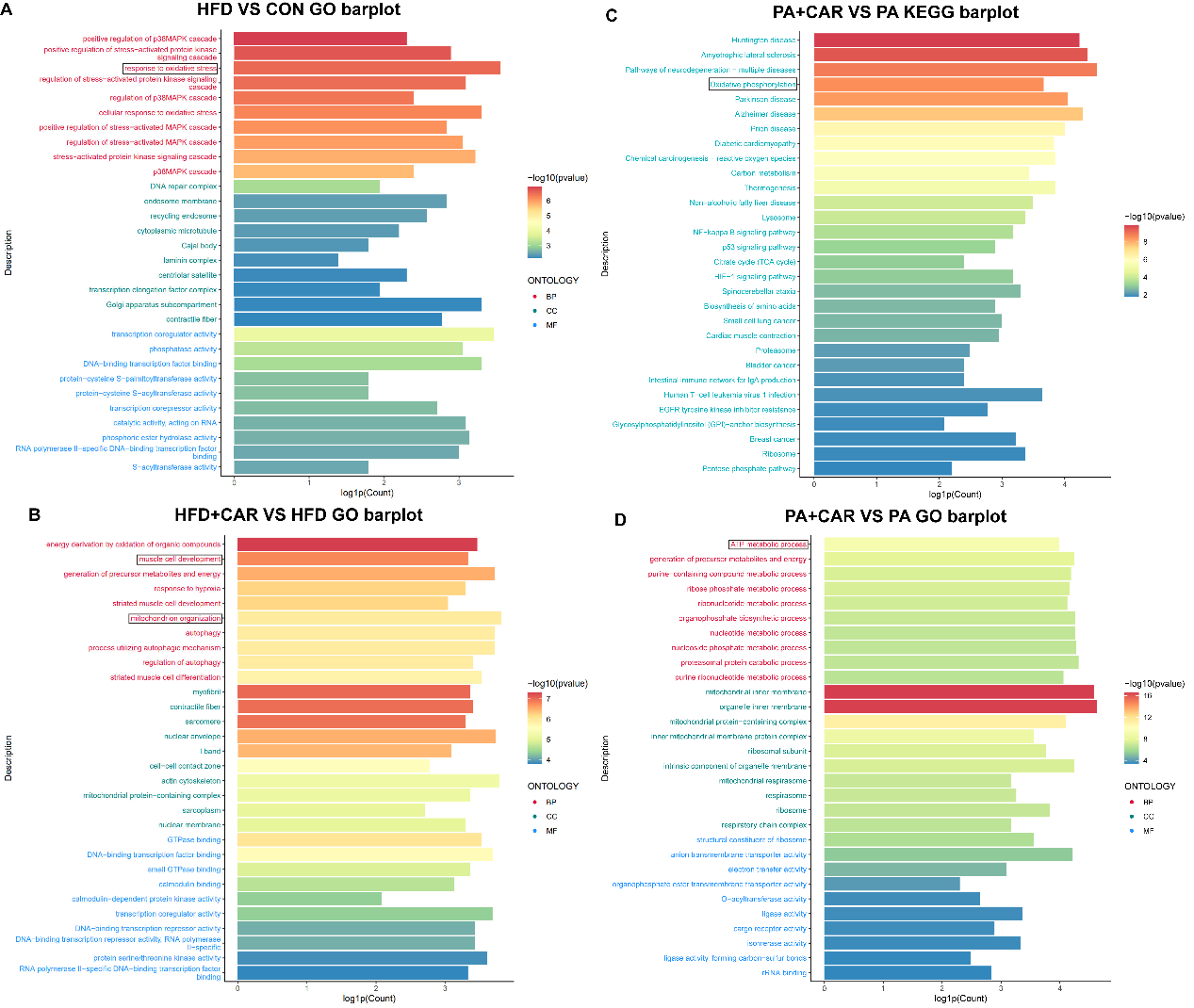


(A) GO enrichment analysis of the control (n=3) and high-fat diet groups (n=3).

(B) GO enrichment analysis of the high-fat diet (n=3) and carnitine-treated groups (n=3).

(C) KEGG enrichment analysis of palmitic acid (n=3) and carnitine-treated groups (n=3).

(D) GO enrichment analysis of the palmitic acid (n=3) and carnitine-treated groups (n=3).

**Figure S6 Heatmap analysis of RNA sequencing results in mice and cells**


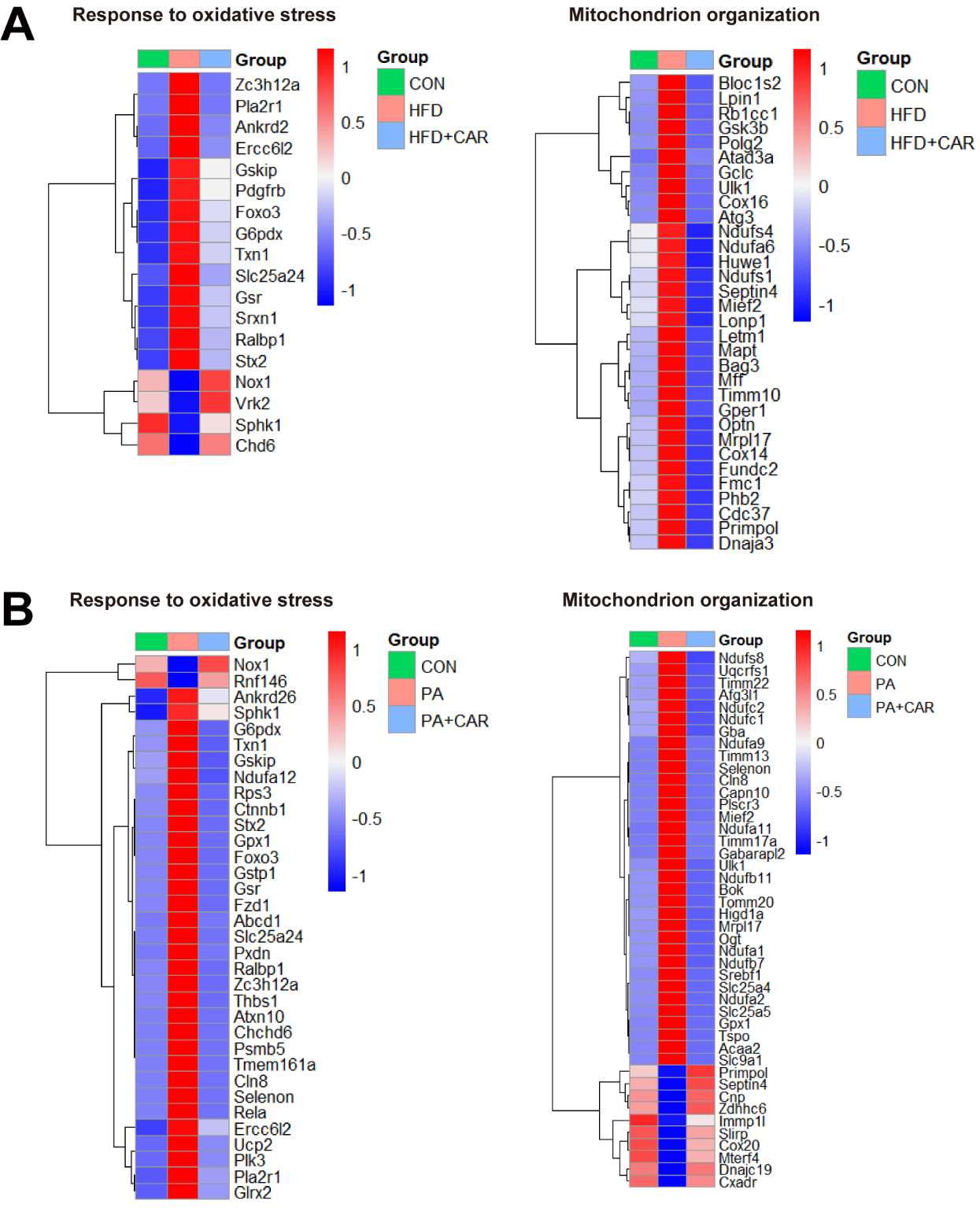


(A) Heatmap of the relative expression levels of oxidative stress-related genes and mitochondrial tissue-related genes in the control (n=3), high-fat diet (n=3) and carnitine-treated groups (n=3), as analyzed via RNA-Seq.

(B) Heatmaps of the relative expression levels of oxidative stress-related genes and mitochondrial tissue-related genes in control (n=3), palmitic acid-treated (n=3) and carnitine-treated (n=3) cells analyzed via RNA-Seq.

**Figure S7 Carnitine supplementation partially rescues the muscle atrophy phenotype in OCTN2 deficiency conditions**


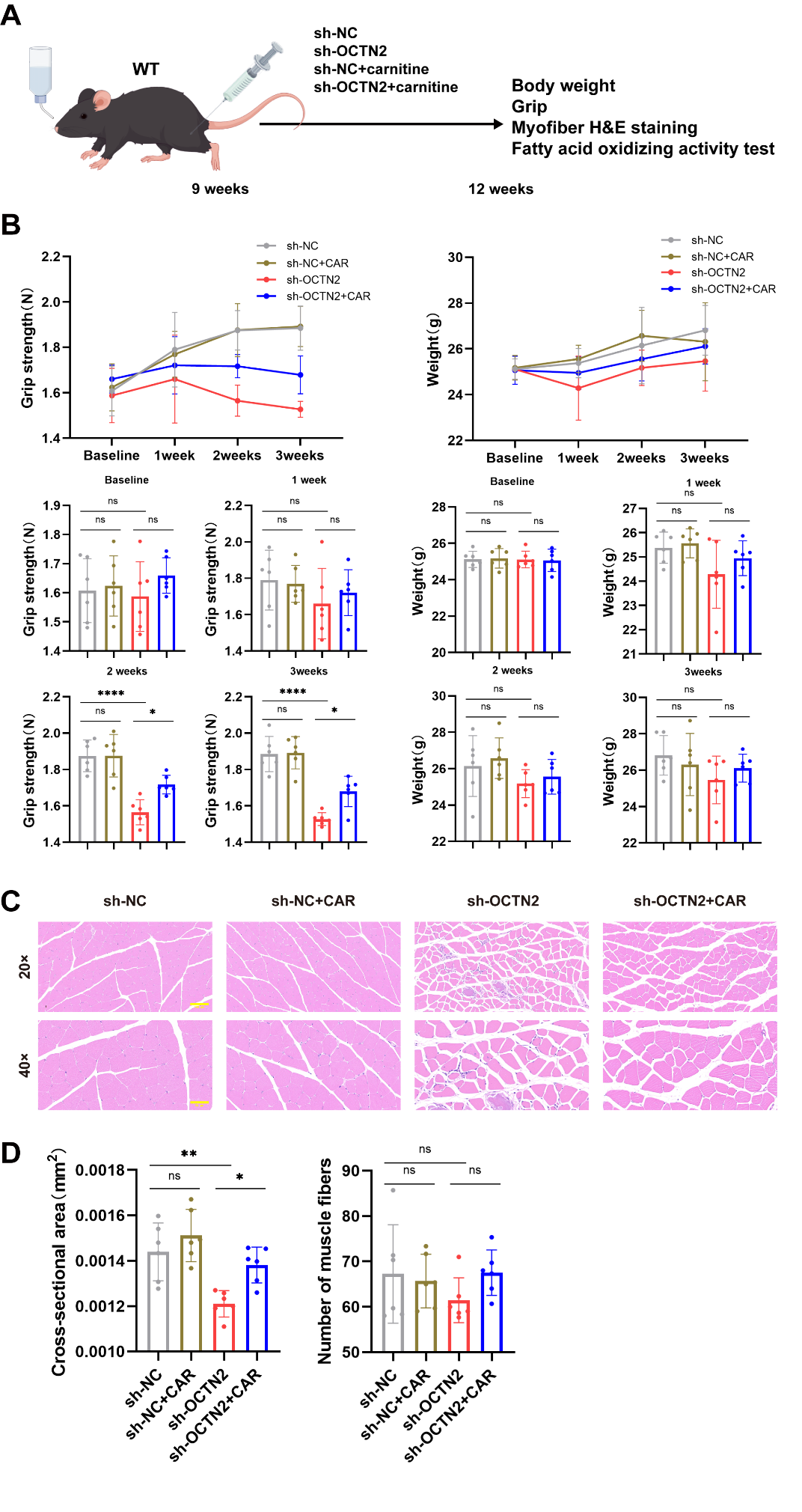


(A) Experimental design model.

(B) Grip strength and body weight of the mice.

(C) H&E staining of the mouse gastrocnemius muscle.

(D) Myofiber cross-sectional area statistics and myofiber number statistics.

**Table S1 Association between serum carnitine and sarcopenia**

| variable | B | SE | Wald | OR_with_CI | P |
| --- | --- | --- | --- | --- | --- |
| (Intercept) | 1.2 | 3.5 | 0.1 | 3.253(0.005~4961.248) | 0.7347 |
| Sex (female/male) | -0.6 | 0.6 | 0.9 | 0.557(0.16~1.801) | 0.3381 |
| Age (years) | 0.1 | 0.0 | 2.9 | 1.07(0.993~1.163) | 0.0885 |
| BMI (kg/m^2^) | -0.1 | 0.1 | 2.1 | 0.873(0.713~1.037) | 0.1488 |
| Serum carnitine (ug/ml) | -0.3 | 0.1 | 6.5 | 0.757(0.599~0.923) | 0.0107 |

The model was adjusted for age, sex and BMI.

**Table S2 Reagents and antibodies**

| Reagents and antibodies | Catalog number | Source |
| --- | --- | --- |
| Anti-CPT1A | ab220789 | Abcam |
| ACADM Polyclonal antibody | Cat No. 55210-1-AP | Proteintech |
| Anti-pYAP | Cat No. 29018-1-AP | Proteintech |
| Anti-TEAD4 | Cat No. 12418-1-AP | Proteintech |
| Anti-MYH | sc-376157 | Santa |
| Anti-GAPDH | ab8245 | Abcam |
| Anti-Solute carrier family 22 member (OCTN2) | ab180757 | Abcam |
| BODIPY 493/503 | D2191 | ThermoFisher Scientific |
| CPT1A Polyclonal antibody | Cat No. 55210-1-AP | Proteintech |
| DEX | HY-46225 | Med Chem Express |
| DOOKU1 | HY-126010 | Med Chem Express |
| Dual Luciferase Reporter Assay Kit | DL101-01 | Vazyme |
| Fatty Acid Oxidation (FAO) Colorimetric Assay Kit | E-BC-K784-M | Elabscience |
| Linoleic acid | HY-N0729 | Med Chem Express |
| Mitochondrial membrane potential detection kit (JC-1) | C2006 | Beyotime Biotechnology |
| MitoSOX Red Mitochondrial Superoxide Indicator | 40778ES50 | Yeasen |
| MSAB | HY-120697 | Med Chem Express |
| OCTN2 Polyclonal antibody | Cat No. 16331-1-AP | Proteintech |
| Oil Red O Staining Kit | C0157S | Beyotime Biotechnology |
| Oleic acid | HY-N1446 | Med Chem Express |
| Palmitic acid | HY-N0830 | Med Chem Express |
| Rhosin HCL | HY-12646 | Med Chem Express |
| Seahorse cell mitochondrial stress testing kit | 103015-100 | Agilent |
| SimpleChIP® Enzymatic Chromatin IP Kit (Magnetic Beads) | 9003S | Cell signaling |
| XMU-MP-1 | HY-100526 | Med Chem Express |
| YAP1 Monoclonal antibody | Cat No. 66900-1-Ig | Proteintech |

**Table S3 Sequence of the primers used for qPCR.**

**Primers Sequence** **(5′-3′)**

**Yap-F** ATTTCGGCAGGCAATACGG

**Yap-R** TGAGACATCCCAGGAGAAGACAC

**OCTN2-F** CACGGAATATCAGGGTCATC

**OCTN2-R** CCGCCAGTAGGAAGCAGT

**ACTB-F** GAGGGAAATCGTGCGTGAC

**ACTB-R** CGCTCGTTGCCAATAGTGAT

**Table S4 Sequence of the primers used for Chromatin immunoprecipitation (ChIP) assay.**


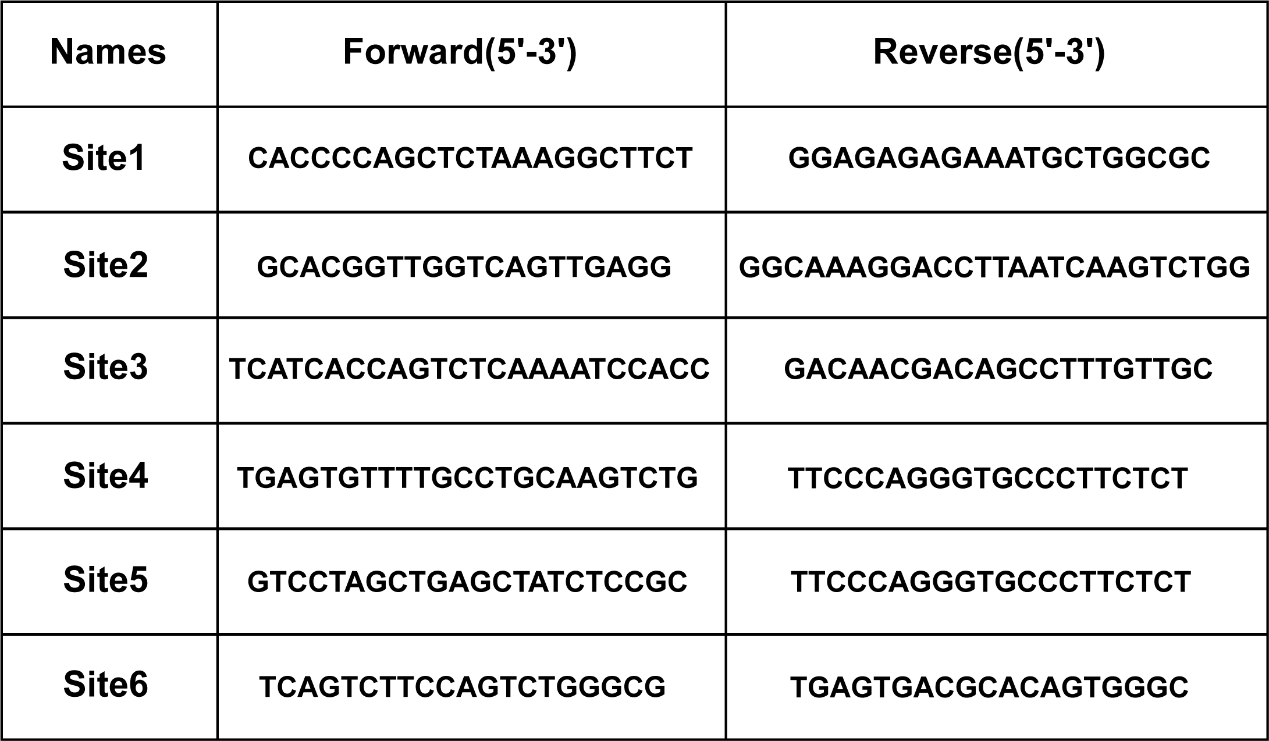


**Table S5 Goutallier classification system**

| Goutallier classification | Definition |
| --- | --- |
| 0 | Normal muscle |
| 1 | Minimal focal or linear fatty infiltration |
| 2 | Less than 50% fat within the muscle |
| 3 | 50% of fat within the muscle |
| 4 | More than 50% fat within the muscle |

**Table S6 Details of the equipment used for MRI and DXA**

| Equipment | Equipment Information |
| --- | --- |
| Magnetic Resonance Imaging (MRI) | Our MRI imaging is performed using a Siemens EQUIPTOM Area 1.5T MR scanning system. Dynamic enhancement scanning was performed using three-dimensional fast low-angle short scanning (3DFLASH): TR 4.5 ms, TE 1.5 ms, inversion angle 10°, layer thickness 1.5 mm, unpitched scanning, FOV 340 mm × 340 mm, and matrix 269 × 384. |
| Dual Energy X-ray Bone Densitometer (DXA) | Our DXA equipment is manufactured by GE Medical Systemes Monterrey. The device model is Prodigy Pro. |


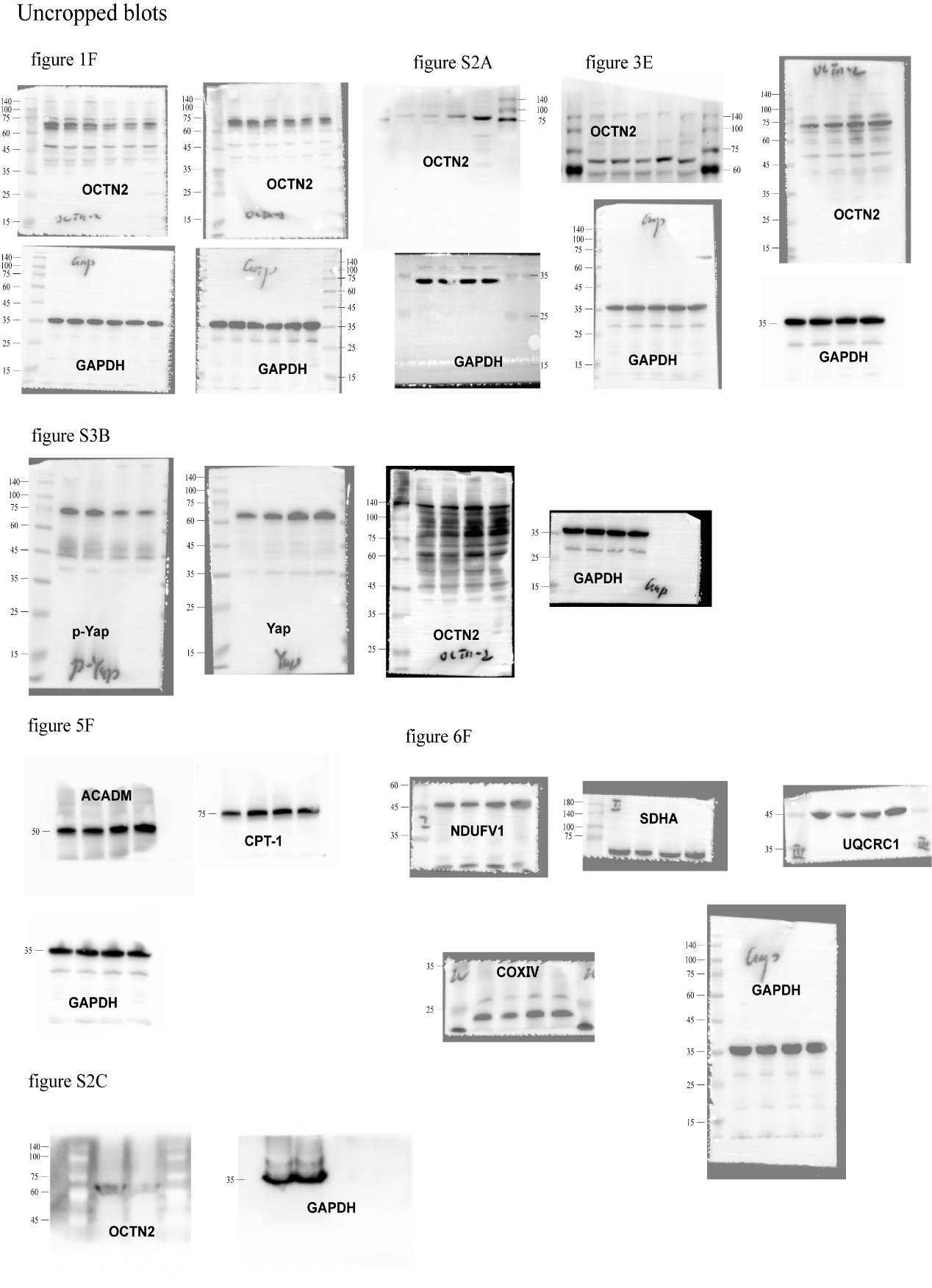


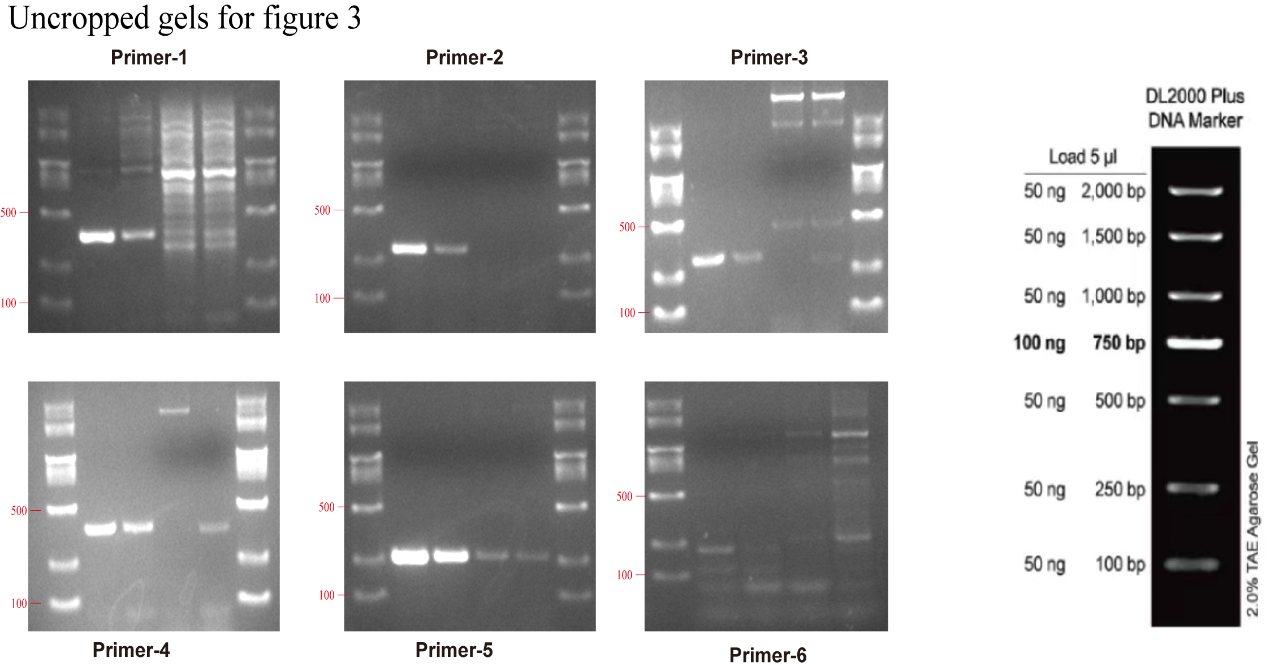

Supplement: Supplementary file 1 — Figure S1: (A) Clinical characteristics of the patients. (B and C) H&E staining of mouse gastrocnemius muscle and measurement of the cross‐sectional area of the sections. (D) The grip strength and body weight of DEX‐treated mice were measured over a two‐week period. Figure S2: (A) Western blot analysis of myotube OCTN2 protein expression at days 0, 1, 3 and 6 of differentiation. The intensity of the OCTN band relative to that of the GAPDH band was quantified via ImageJ software (n = 3). (B) mRNA expression of OCTN2 was assessed by RT–qPCR at different stages of C2C12 differentiation. (C)Western blot analysis of the protein levels in the expression of shOCTN2‐Injected Mice and shNC‐Injected Mice. (D) Fatty acid oxidising activity in muscle of shOCTN2‐injected mice and shNC‐injected mice. Figure S3: (A) Experimental design model. (B) Western blotting was used to analyse the expression levels of phospho‐YAP, YAP and OCTN2 in C2C12 myotubes treated with tension force for 6 h or not and then cultured for 18 h. RT–qPCR analysis of the expression levels of YAP and OCTN2 in C2C12 myotubes treated with tension force for 6 h or not and then cultured for 18 h. (C) RT–qPCR analysis of OCTN2 mRNA expression under tension stimulation by Yap silencing. Figure S4: (A) Schematic diagram of modelling of mice injected with sh‐OCTN2 adenovirus for swimming exercise. (B) Schematic diagram of modelling of XMU‐treated DEX‐induced myasthenia gravis mice. Figure S5: (A) GO enrichment analysis of the control (n = 3) and high‐fat diet groups (n = 3). (B) GO enrichment analysis of the high‐fat diet (n = 3) and carnitine‐treated groups (n = 3). (C) KEGG enrichment analysis of palmitic acid (n = 3) and carnitine‐treated groups (n = 3). (D) GO enrichment analysis of the palmitic acid (n = 3) and carnitine‐treated groups (n = 3). Figure S6: Heatmap analysis of RNA sequencing results in mice and cells. (A) Heatmap of the relative expression levels of oxidative stress‐related genes and mitochondrial [file JCSM-16-e70052-s001.docx]
